# Developing Consumer Consensus on Remote Assessment and Management of Physical Function in Older Adults (RAMP): International Modified Delphi Process

**DOI:** 10.2196/75791

**Published:** 2026-02-06

**Authors:** Elsa Dent, Christopher Hurst, Jack Dalla Via, Jackson J Fyfe, Paul Jansons, Eleanor J Hayes, Gary Skinner, Marc Sim, Mylene Aubertin-Leheudre, Sabine Britting, Fanny Buckinx, Gavin Connolly, Ruth Dignam, Lora Giangregorio, Jennifer R A Jones, Pauline Kelly, Robert Kob, Suzanne N Morin, Girish Nandakumar, Lucas B R Orssatto, Maria Pearson, Daniel Pinto, Esmee M Reijnierse, Catherine M Said, Mohamed Salem, Vina PS Tan, Rosanna Tran, Jesse Zanker, Robin M Daly, David Scott

**Affiliations:** 1 Division of Health University of Waikato Hamilton New Zealand; 2 AGE Research Group, Translational and Clinical Research Institute, Faculty of Medical Sciences Newcastle University Newcastle upon Tyne United Kingdom; 3 NIHR Newcastle Biomedical Research Centre, Newcastle upon Tyne Hospitals NHS Foundation Trust, Cumbria Northumberland Tyne and Wear NHS Foundation Trust and Faculty of Medical Sciences Newcastle University Newcastle upon Tyne United Kingdom; 4 Institute for Physical Activity and Nutrition (IPAN), School of Exercise and Nutrition Sciences Deakin University Burwood Australia; 5 Nutrition & Health Innovation Research Institute, School of Medical and Health Sciences Edith Cowan University Joondalup Australia; 6 School of Clinical Sciences at Monash Health Monash University Clayton Australia; 7 Department of Sport, Exercise and Rehabilitation Northumbria University Newcastle Upon Tyne United Kingdom; 8 School of Medicine The University of Western Australia Perth Australia; 9 Département des Sciences de l'Activité Physique, Faculté des Sciences, Groupe de Recherche en Activité Physique Adaptée (GRAPA) Université du Québec à Montréal Montréal, QC Canada; 10 Centre de Recherche de l'Institut Universitaire de Gériatrie de Montréal (CRIUGM) Montréal, QC Canada; 11 Institute for Biomedicine of Aging Friedrich-Alexander-Universität Erlangen-Nürnberg Nuremberg Germany; 12 Research Unit in Public Health, Epidemiology and Health Economics University of Liège Liège Belgium; 13 Metabolism and Healthy Aging Research Center (MHARC), Division of Endocrinology, Diabetes, and Metabolism, Department of Medicine Cedars-Sinai Medical Center Los Angeles, CA United States; 14 IPAN Consumer Network Deakin University Geelong Australia; 15 Schlegel-UW Research Institute for Aging University of Waterloo Waterloo, ON Canada; 16 Department of Kinesiology and Health Sciences University of Waterloo Waterloo, ON Canada; 17 Physiotherapy Department The University of Melbourne Parkville Australia; 18 Physiotherapy Department, Division of Allied Health Austin Health Heidelberg Australia; 19 Institute of Breathing and Sleep Heidelberg Australia; 20 Division of General Internal Medicine, Department of Medicine McGill University Montréal, QC Canada; 21 Department of Physiotherapy, Manipal College of Health Professions Manipal Academy of Higher Education Manipal India; 22 Centre for Sensorimotor Performance, School of Human Movement and Nutrition Sciences, Faculty of Health, Medicine and Behavioural Sciences The University of Queensland Brisbane Australia; 23 Maria Pearson Coaching Havelock North New Zealand; 24 Department of Physical Therapy Marquette University Milwaukee, WI United States; 25 Department of Nutrition and Dietetics Faculty of Health, Sport and Physical Activity Amsterdam University of Applied Sciences Amsterdam The Netherlands; 26 Physiotherapy Western Health St Albans Australia; 27 Australian Institute for Musculoskeletal Science (AIMSS) The University of Melbourne and Western Health St Albans Australia; 28 Geriatrics Active Ageing and Community Care Ministry for Health and Active Ageing Valletta Malta; 29 Exercise and Sports Science Programme, School of Health Sciences Universiti Sains Malaysia Kubang Kerian Malaysia; 30 Rehabilitation and Aged Care Services Northern Sydney Local Health District Sydney Australia; 31 Department of Medicine - Royal Melbourne Hospital The University of Melbourne Parkville Australia

**Keywords:** aged, Delphi technique, exercise, remote consultation, digital health, physical function

## Abstract

**Background:**

Remote health care delivery, including the use of digital health interventions, is emerging as a tool for assessing and managing physical function, but its design and implementation often overlook the needs and preferences of older adult end users.

**Objective:**

The primary aim of this modified Delphi process was to develop consumer consensus on preferences for remote assessment and management of physical function in older adults.

**Methods:**

Research and consumer experts of the Remote Assessment and Management of Physical Function in Older Adults (RAMP) Working Group co-developed the Round 1 Delphi survey, which was advertised to consumers (adults aged ≥60 years) via international clinical and research networks and social media between August and November 2023. The online survey presented 23 Delphi statements for which respondents reported their level of agreement using an 11-point Likert scale (0-10; scores ≥7 indicated agreement). Statements were classified as having “strong agreement” and achieving consensus if ≥80% of participants indicated agreement. Statements classified as having “moderate” (70%-80% of participants indicated agreement) or “low” (<70% of participants indicated agreement) agreement were revised or rejected. Revised statements were presented to participants in Round 2 (January to February 2024), and the final consensus statements were consolidated into recommendations.

**Results:**

A total of 654 consumers (75.7% female) with a mean age of 69.0 (SD 6.0) years from 15 countries (5 continents) were included in analyses in Round 1. Of 23 statements, 13 achieved consensus, with the strongest agreement observed for statements relating to the importance of physical function for quality of life and performing activities of daily living (6 statements; agreement 97.6%-99.5%). Two statements regarding privacy and security concerns when using technology (agreement 20.8%) and the inability to perform physical function assessments or exercise at home (agreement 15.5%) were rejected with low agreement. The remaining 8 statements (agreement 49.5%-79.5%) were modified into 7 new statements for the Round 2 survey, which was completed by 526 (80.4%) respondents from Round 1. Five of seven Round 2 statements were accepted with strong agreement (agreement 80%-82.7%), including the importance of addressing personal preferences for self- versus clinician-led remote interventions, group versus individual exercise, and availability of necessary resources (eg, technology and exercise equipment).

**Conclusions:**

Eighteen statements achieved consensus and were translated into 7 recommendations highlighting that older adults recognize physical function as a health priority, would value more information about it, and are willing to participate in remote assessment and management interventions (including via digital health) to maintain or improve it. These recommendations also reinforce that interventions should be easily accessible and meet individual preferences of consumers.

## Introduction

Maintaining physical function is crucial for older adults to live independently and maintain a good quality of life [1]. Depending on the task examined, up to 50% of older adults may report difficulty with physical function, and more than 10% use walking aids, with an increasing prevalence of functional limitation in older age (eg, ≥80 years) [2]. Older adults with functional limitations are half as likely to engage with their communities, family, and friends [3] and have significantly increased health care costs compared to those without functional limitations [4]. However, assessment and management of physical function receive limited attention in many clinical settings [5-7]. While multimodal exercise is recommended for improving physical function [8], older adults report that lack of access to interventions, facilities, and relevant health care professionals are barriers to participation [9].

Remote health care services enable patient assessment and monitoring without physical co-location [10] and, especially since the COVID-19 pandemic, have been commonly used by older adults [11]. Emerging evidence suggests digital health interventions using technologies such as telephone calls, videoconferencing, wearable devices, and web applications are feasible and effective for supporting older adults to maintain or improve their physical function [12-14]. However, both consumers (ie, older adult end users) and health care professionals face barriers to engagement with digital health interventions, such as low digital literacy, lack of equipment, and unsatisfactory social interactions [15,16]. Furthermore, the design and implementation of digital health technologies for remote care have largely failed to consider the perceptions and experiences of consumers in their design and implementation, impeding large-scale sustained adoption [17].

The Delphi method, a rigorous consensus-building approach [18], is effective for developing recommendations for the delivery of care related to maintaining and improving physical function in older adults based on the insights of both experts and consumers (ie, older adults themselves) [19,20]. Incorporating consumer participation in consensus-building processes helps ensure that subsequent recommendations address end users’ priorities, maximizing the potential for wide adoption and adherence among consumers in health care and community settings [21]. However, no consumer-focused consensus Delphi process has explored the priorities, acceptability, enablers, and barriers for remote assessment and management of physical function from older adults’ perspectives, and there are no consensus guidelines on the delivery of remote care in older adult populations. Thus, this study aimed to develop consumer-informed recommendations for remote assessment and management of physical function in older adults via a modified Delphi process.

## Methods

### Study Design and Population

The Remote Assessment and Management of Physical Function in Older Adults (RAMP) International Consumer Delphi Process was a modified (2-round) Delphi study. Eligible participants were aged 60 years and older with internet access, residing in any country, and able to complete the survey in the English language.

RAMP was advertised to potential participants via email (consumer mailing lists of the RAMP Working Group), direct invitations (investigator-led conversations with consumers), and social media (Facebook [Meta Platforms, Inc] advertisements; posts on X [X Corp] and LinkedIn [LinkedIn Corp]) between August and November 2023.

### Delphi Process

This study’s methodology adhered to that of a previous modified Delphi process where 2 rounds, as demonstrated by common practice in previous Delphi studies [22,23], were sufficient to reach consensus on sarcopenia management [19]. Multimedia Appendix 1 summarizes the study timeline. The Round 1 survey (Multimedia Appendix 2) was co-developed by RAMP Working Group research experts (DS, CH, JF, PJ, and RMD) and consumer experts (RD and PK) and included questions on demographics, health status (including the SARC-F questionnaire [24]), and health care experiences, plus 23 Delphi statements related to physical function and its assessment and management. The first 13 statements relate to physical function generally, while the latter 10 statements refer to remote provision of health care related to physical function. Respondents were asked to report their level of agreement with each Delphi statement via an 11-point Likert scale (0=Strongly disagree, 10=Strongly agree) and could optionally provide a free-text comment explaining their response to each statement.

Round 1 survey responses were analyzed by DS, and the results and proposed Round 2 survey were shared with RAMP Working Group members for feedback. In January 2024, an invitation to participate in the Round 2 survey, which included seven new or modified statements, was emailed to respondents who completed Round 1, along with a summary of the Round 1 results and an explanation of decisions taken in developing the Round 2 statements (Multimedia Appendix 3). Further details on reasons for low or moderate agreement to Round 1 statements and decisions taken are provided in Multimedia Appendix 4. Upon completion of the Round 2 survey (Multimedia Appendix 5), DS analyzed the results, and ED and DS initially developed the final recommendations. Finally, this manuscript, including the developed recommendations, was revised (2 rounds of revisions) and approved by RAMP Working Group members.

### Statistical Analyses

Survey data were assessed for completeness, and all respondents who completed ≥50% of survey questions were included in analyses. Where duplicate responses were identified, the most complete and/or first response was included. Descriptive characteristics were reported as frequencies or percentages for categorical variables and means and SDs or medians and IQR for continuous variables.

Participants with a response ≥7 out of 10 were considered to have agreed with a given statement, and the level of consensus was determined by the proportion of participants who agreed. Round 1 and 2 Delphi statements were classified as having “strong agreement” if ≥80% of participants responded with a score ≥7 out of 10 [19]. These statements were considered to have achieved consensus and were not further modified. In Round 1 only, statements with “moderate” (70%-80% of participants responded ≥7) or “low” (<70% of participants responded ≥7) agreement were revised or rejected. DS reviewed the associated free-text comments and revised the statements based on common reasons for lack of agreement. The revised statements were shared with the RAMP Working Group for approval. In Round 2, all statements that did not achieve “strong agreement” were rejected. All analyses were performed using SPSS Statistics 28 (IBM Corp).

### Ethical Considerations

The study was approved by the Deakin University Human Ethics Advisory Group (Reference number: HEAG-H 111_2023) and complied with the Declaration of Helsinki. Potential participants were directed to an online plain language statement defining physical function (ie, “the ability of a person to perform everyday activities”), as well as the study aims and methods. Potential participants provided an electronic signature confirming their informed consent. Those who consented were subsequently emailed a link to the Round 1 Delphi survey (hosted by Qualtrics XM). Participants who completed the Round 1 survey were invited to participate in the Round 2 survey between January and February 2024. Participants who consented but had not completed the Round 1 and 2 surveys were sent a reminder 2 weeks prior to the closing date. Participation was voluntary, and respondents did not receive any form of reimbursement.

## Results

### Round 1

A total of 861 complete consent forms were received, and the Round 1 Delphi survey subsequently received 716 responses during the Round 1 survey period. Of these responses, 50 (7%) completed less than 50% of the survey, and 12 (2%) were duplicate responses. Thus, 654 of 861 (76%) consented consumers were included in the Round 1 survey analyses. Most of the included respondents (n=644, 98%) completed the entire Round 1 survey, while 10 respondents rated their agreement with the first 13 statements only. Respondents’ mean age was 69.0 (SD 6.0) years, and three-quarters were female (Table 1). Respondents were residing in 15 countries across 5 continents (Australia, Africa, Asia, Europe, and North America), although the majority (81.5%) were from Australia and the United Kingdom. Three-quarters of respondents had completed higher education and around 70% were retired. Over 87% rated their health as good to excellent, but more than 70% perceived their current physical function was “somewhat worse” or “much worse” than when they were 40 years old. The SARC-F instrument demonstrated that approximately 54% of respondents had some functional limitation, and around one-quarter had experienced at least one fall in the past year (Multimedia Appendix 6).

**Table 1:** Demographic characteristics of Remote Assessment and Management of Physical (RAMP) participants in Round 1.

Characteristic		All respondents (n=654)
Age (years), mean (SD)		69 (6.0)
Sex (female), n (%)		495 (75.7)
**Country of residence, n (%)**		
	Australia	281 (43.0)
	United Kingdom	252 (38.5)
	Canada	32 (4.9)
	Ireland	22 (3.4)
	United States	20 (3.1)
	New Zealand	15 (2.3)
	Germany	7 (1.1)
	Singapore	7 (1.1)
	Malta	6 (0.9)
	India	5 (0.8)
	Netherlands	3 (0.5)
	Belgium	1 (0.2)
	France	1 (0.2)
	Georgia	1 (0.2)
	South Africa	1 (0.2)
**Highest level of education, n (%)**		
	Infants or primary school	3 (0.5)
	Secondary or high school	154 (23.5)
	University, college, or other higher education	497 (76.0)
**Employment status, n (%)**		
	Employed full-time	67 (10.2)
	Employed part-time	90 (13.8)
	Home duties	3 (0.5)
	Pension	18 (2.8)
	Retired	462 (70.6)
	Student	3 (0.5)
	Unemployed	11 (1.7)
**General health, n (%)**		
	Excellent	89 (13.6)
	Very good	255 (39.0)
	Good	228 (34.9)
	Fair	67 (10.2)
	Poor	15 (2.3)
**Physical function now compared with that at age 40 years, n (%)**		
	Much better	18 (2.8)
	Somewhat better	44 (6.7)
	Neither better nor worse	117 (17.9)
	Somewhat worse	348 (53.2)
	Much worse	127 (19.4)
SARC-F score, median (IQR)		1 (0-2)

Less than one-third of respondents reported that a health care professional had started a conversation with them about their physical function in the past 5 years, while 38% had initiated a conversation themselves (Table 2). More than 60% had sought information on physical function from sources other than a health care professional. Around 40% of respondents reported having ever completed a physical function assessment with a health care professional, and almost 50% had commenced a supervised exercise program aimed at improving their physical function. The majority of respondents (>60%) had commenced an unsupervised exercise program aimed at improving their physical function.

**Table 2:** Health care experiences related to physical function for Remote Assessment and Management of Physical (RAMP) participants in Round 1 (n= 654).

Question	Yes, n (%)	No, n (%)	Don’t Know, n (%)
In the past five years, have you started a conversation with a health professional (eg, doctor, physiotherapist, and nurse) about your physical function (eg, asking how you can continue to stay independent as you get older, or why you might not be as strong as you were when you were younger)?	245 (37.5)	399 (61)	9 (1.4)
In the past 5 years, has a health professional (eg, doctor, physiotherapist, and nurse) started a conversation with you about your physical function?	209 (32)	436 (66.7)	7 (1.1)
Have you ever tried to find information about physical function from sources other than a health professional (eg, by asking a friend or family member, visiting a website, or reading a book or magazine)?	395 (60.4)	255 (39)	3 (0.5)
Have you ever completed a physical function test (eg, walking speed test, hand grip strength test, and chair stand test) under the supervision of a health professional (eg, where a health professional asked you to perform a specific test while under their supervision to determine whether your physical function was poor)?	270 (41.3)	376 (57.5)	7 (1.1)
Have you ever completed a physical function test (eg, walking speed test, hand grip strength test, and chair stand test) while NOT under the supervision of a health professional (eg, performing a specific test designed to determine whether your physical function is poor after you read or viewed instructions in a document or online)?	101 (15.4)	543 (83)	9 (1.4)
Has a health professional ever prescribed you an exercise program aimed at improving your physical function?	315 (48.2)	333 (50.9)	5 (0.8)
Have you ever commenced an exercise program aimed at improving your physical function while NOT under the supervision of a health professional (eg, an exercise program that you created for yourself, with or without the help of a friend or family member, or using a website/book/magazine)?	402 (61.5)	245 (37.5)	6 (0.9)

**Figure 1:**
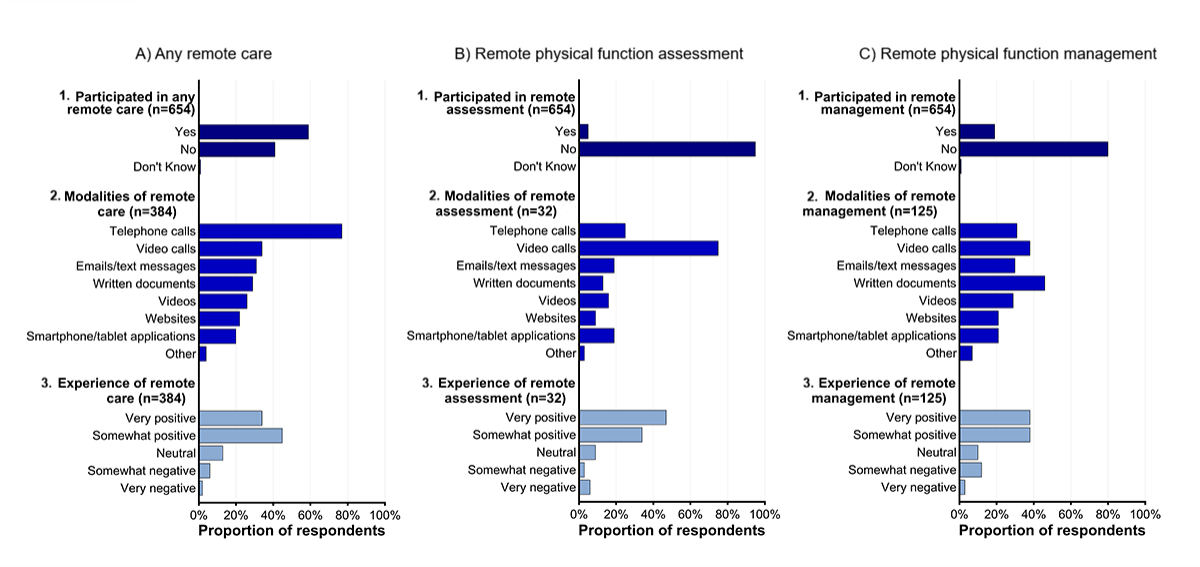
Remote health care experiences of RAMP participants in Round 1, related to (A) any type of remote care, (B) remote care to assess physical function, and (C) remote care to manage physical function. Sublabels (1) describe the proportion of respondents who reported having participated in that type of remote care, (2) report the modalities by which respondents engaged with that type of remote care, and (3) report the perceived experience of respondents participating in that type of remote care. Questions 2 and 3 were only asked to participants who responded yes to having participated in that type of remote care. For question 2, participants could choose multiple responses, so the total does not equal 100%.

Over 58% of respondents had participated in a remote health care service, while less than 5% had participated in a remote physical function test, and less than 20% had engaged in remote physical function management such as an exercise program (Figure 1). For respondents who had participated in any form of remote care, telephone calls were most commonly used (77%), with video calls and emails/text messages each used by over 30% of respondents. For respondents who had participated in a remote physical function assessment, video calls were most commonly used (75%), followed by telephone calls (25%). For respondents who had participated in a remote treatment for their physical function, written documents (eg, flyers, brochures, magazines, and books) were most common (46%), followed by video calls (38%) and telephone calls (31%). Regardless of the type of remote care, respondents generally reported positive experiences; 75%-81% rated their experience as somewhat or very positive.

Only 5% of respondents reported that they would not be willing to participate in remote physical function tests and treatments (Multimedia Appendix 7), with optional comments indicating that this was due to concerns regarding lack of supervision, safety, lack of necessary technologies and technological familiarity, data privacy, hearing and visual problems, potential costs, and insufficient space in the home. Amongst those willing to participate in such interventions, videos (eg, on a website or DVD) were the most preferred delivery method (78%). Approximately 4% were willing to use other approaches; free-text comments indicated that these could include digital voice assistants, wearable devices, webinars, smart televisions, and wall charts. The most common perceived positives of remote tests and treatments, selected by over 80% of respondents, were the convenience of not needing to travel to appointments and the flexibility to perform assessments and exercises when suitable. Some (<4%) respondents nominated other positives in free-text comments, including avoiding body shaming or embarrassment when exercising, self-motivation, empowerment and autonomy for managing exercise, and better use of health care professionals’ time. Regarding negatives of remote care, the lack of personalized guidance during exercises was reported by half (51%) of respondents, and concerns regarding the potential ineffectiveness of interventions and a lack of social interaction and motivation were each reported by more than 40%. Approximately 14% of respondents cited other potential negatives, including costs and/or lack of access to necessary equipment and technological devices, lack of confidence in performing exercises correctly, low motivation to exercise, lack of engagement with programs and health care professionals, and insufficient space to perform exercises at home.

**Figure 2:**
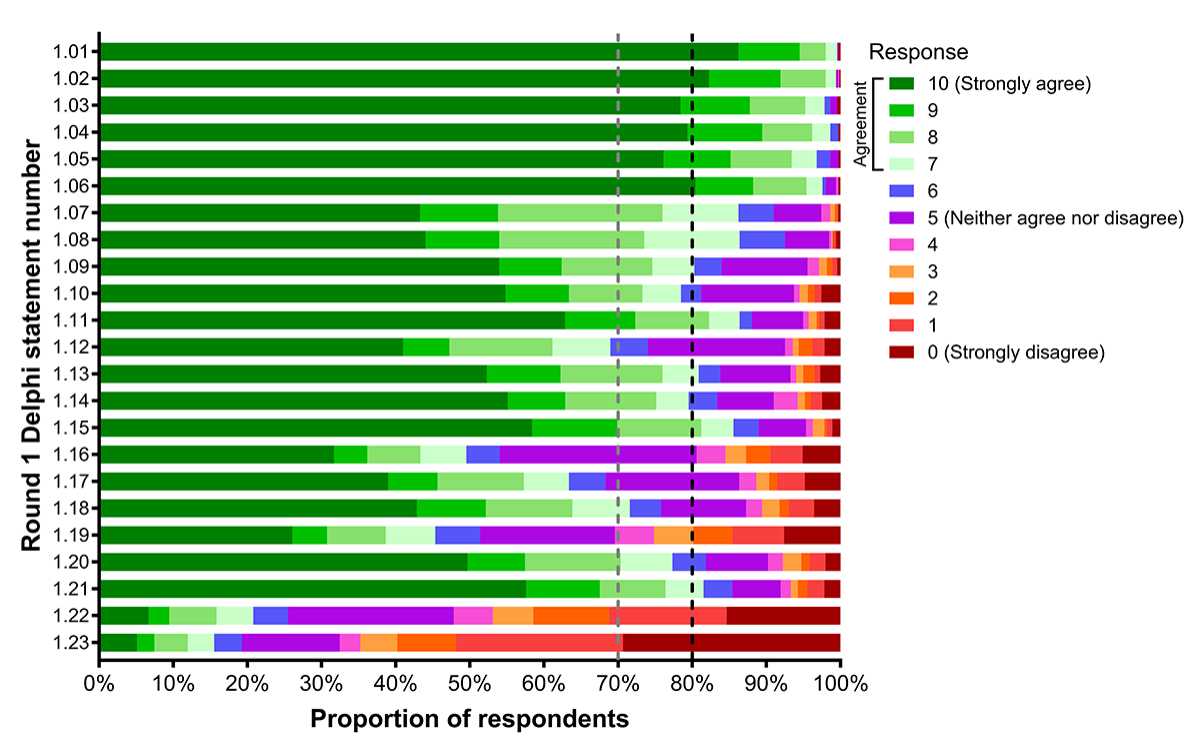
Representation of participants’ level of agreement (rated on an 11-point Likert scale; 0-10) with each of 23 Round 1 Delphi statements. n=654 for statements 1.01-1.13, n=644 for statements 1.14-1.23. Participants with a response ≥7 were considered to have agreed with the statement. Statements with strong agreement (≥80%; black dotted line) were accepted. Statements with 70%-<80% agreement (grey dotted line) were considered to have moderate agreement. Statements with <70% agreement were considered to have low agreement.

Figure 2 and Table 3 present respondents’ reported agreement with the 23 Delphi statements from Round 1. Statements related to the importance of physical function for maintaining overall quality of life, participating in activities in the community and home, and engaging with family and friends (Statements 1.01-1.06) had the highest proportion (97%-100%) of respondents who reported agreement (response ≥7 out of 10) in this round and were accepted. Consensus was also achieved that poor physical function can be prevented and reversed (Statements 1.07 and 1.08; both 86% agreement) and that respondents would discuss with their health care professionals if concerned about their physical function (Statement 1.09; 80% agreement). There was moderate agreement that respondents would like access to information on how to test their physical function (Statement 1.10; 78% agreement) and low agreement that having better access to information would assist in conversations with health care professionals (Statement 1.12; 69% agreement). However, there was strong agreement that respondents would like access to information on how to improve their physical function (Statement 1.11; 86% agreement) and that having better access to information would assist in managing their physical function independently (Statement 1.13; 81% agreement).

Regarding remote provision of health care, almost 80% of respondents agreed that they would be willing to participate in remote physical function tests, but this statement (Statement 1.14) did not meet the criterion for consensus. There was strong agreement that physical function tests would be safe to perform without direct supervision (Statement 1.15; 86% agreement) but only low agreement that respondents would be willing to participate in a remote exercise program that was always (Statement 1.16; 50% agreement) or sometimes (Statement 1.17; 63% agreement) supervised, and moderate agreement that they would be willing to participate in an unsupervised exercise program (Statement 1.18; 72% agreement). There was also low or moderate agreement that respondents would be happy to participate in a remote exercise program in a group setting (Statement 1.19; 45% agreement) or individually (Statement 1.20; 77% agreement). Finally, low proportions of respondents agreed that they would be concerned about their privacy and security when using technology to participate in remote care (Statement 1.22; 21% agreement) or that remote physical function tests or exercise programs would be difficult to perform in their home (Statement 1.23; 16% agreement).

**Table 3:** List of Round 1 Delphi statements with levels of agreement and outcome decisions.

Statement number	Statement	Agreement (%)^a^	Agreement rating^b^	Outcome
1.01	Having good physical function is important to the overall quality of life of older adults	99.5	Strong agreement	Accept
1.02	Having good physical function is important for activities involving moving around the community (eg, going shopping or to a restaurant or cafe, visiting your neighbors, friends and family, or the doctor)	99.4	Strong agreement	Accept
1.03	Having good physical function is important for participating in activities with family and friends (eg, playing with grandchildren)	97.9	Strong agreement	Accept
1.04	Having good physical function is important for participating in activities like work, household duties (eg, cooking, cleaning, and gardening), and volunteering	98.6	Strong agreement	Accept
1.05	Having good physical function is important for participating in hygiene activities (eg, showering, dressing, and using the toilet)	96.8	Strong agreement	Accept
1.06	Having good physical function is important for participating in exercise (eg, walking, swimming, dancing, golf, and other types of physical activity)	97.6	Strong agreement	Accept
1.07	It is possible to slow down or prevent poor physical function that occurs as we get older	86.2	Strong agreement	Accept
1.08	If someone already has poor physical function, it is possible to improve it	86.4	Strong agreement	Accept
1.09	If I was concerned about my physical function, I would discuss it with my health professional	80.3	Strong agreement	Accept
1.10	I would like access to information about how to test my physical function myself to determine if it is poor	78.4	Moderate agreement	Modify statement for Round 2
1.11	I would like access to information about things that I can do myself to improve my physical function	86.4	Strong agreement	Accept
1.12	Having better access to information on physical function would help me to have conversations about this with health professionals	69.0	Low agreement	Modify statement for Round 2
1.13	Having better access to information on physical function would help me to take care of my own physical function	80.9	Strong agreement	Accept
1.14	I would be willing to participate in remote tests of my physical function (eg, on a video call with a health professional, or by myself using written instructions and/or video demonstrations provided to me)	79.5	Moderate agreement	Modify statement for Round 2
1.15	I am confident that it would be safe for me to perform physical function tests at home without direct supervision by a health professional if I was provided with instructions (eg, written information or video demonstrations)	85.6	Strong agreement	Accept
1.16	I would be willing to participate in a remote exercise program to improve my physical function if it was ALWAYS supervised (eg, exercising while on a live video call with a health professional for all exercise sessions)	49.5	Low agreement	Modify statement for Round 2
1.17	I would be willing to participate in a remote exercise program if it was SOMETIMES supervised (eg, exercising on a live video call with a health professional for some exercise sessions, but exercising by myself unsupervised using instructions provided by the health professional for other sessions)	63.4	Low agreement	Modify statement for Round 2
1.18	I would be willing to participate in a remote exercise program if it was NOT supervised (eg, exercising by myself unsupervised using instructions provided by a health professional)	71.6	Moderate agreement	Modify statement for Round 2
1.19	If I was to participate in a remote exercise program I would be happy to do so with a group (eg, exercising by myself at home but while on a video call with other people like me who are also exercising at home, with or without the supervision of a health professional)	45.3	Low agreement	Modify statement for Round 2
1.20	If I was to participate in a remote exercise program to improve my physical function, I would be happy to do so alone without other people like me involved in the exercise sessions (eg, exercising by myself at home with or without supervision by a health professional)	77.3	Moderate agreement	Modify statement for Round 2
1.21	I would be comfortable using technology (eg, computers, smartphones, and tablets) to participate in remote tests and treatments for my physical function	81.5	Strong agreement	Accept
1.22	I would be concerned about the privacy and security of my personal information when participating in remote tests and treatments for physical function using technology (eg, computer, smartphone, or tablet)	20.8	Low agreement	Reject
1.23	Remote physical function tests or exercise programs would be difficult to perform in my home (eg, because there is limited space)	15.5	Low agreement	Reject

^a^Proportion of participants who rated statement ≥7 out of 10.

^b^Statement classification based on the following criteria: Strong agreement (>80% of respondents rated statement ≥7 out of 10); Moderate agreement (70% to 80% of respondents rated statement ≥7 out of 10); Low agreement (<70% of respondents rated statement ≥7 out of 10).

### Round 2

Following Round 1, 13 of 23 statements were accepted to have achieved consensus with strong agreement. It was determined that statements 1.22 and 1.23 had such low agreement that they should be rejected rather than modified and presented again in Round 2. Other statements that achieved moderate or low agreement (Statements 1.10, 1.12, 1.14, 1.16, 1.17, 1.18, 1.19, and 1.20) were modified. In brief, Statements 1.10, 1.12, and 1.14 were revised into Statements 2.10, 2.12, and 2.14, respectively. Statements 1.16, 1.17, and 1.18, which covered similar concepts regarding remote exercise supervision, were merged into a single revised statement (Statement 2.16). Statements 1.19 and 1.20 covered similar concepts regarding preferences for participating in remote exercise individually or in a group setting and were revised into a single statement (Statement 2.19). Finally, based on common themes identified in free-text responses to several statements across Round 1, 2 new statements were introduced for Round 2: Statement 2.24 explored the importance for consumers that remote physical function tests have been demonstrated to be safe and accurate, and Statement 2.25 explored the importance of having access to necessary information and resources to support participation in remote exercise programs. Multimedia Appendices 3 and 4 provide further details on the development of Round 2 Delphi statements.

Among participants who completed Round 1, 526 (80%) completed Round 2. No notable differences were observed between the Round 2 and Round 1 respondents, respectively, for age (mean = 69.3, SD 5.7 vs 69.0, SD 6.0 years) or SARC-F score (median 1.0, IQR 0.0-2.0 vs median 1.0, IQR 0.0-2.0). In Round 2, respondents were asked to rate their physical function compared with when they completed the Round 1 survey (mean interval between survey completions was 117.2, SD 21.5 days). The majority (68%) reported that their physical function was neither better nor worse, while 13% reported their physical function was somewhat or much better, and 19% reported that it was somewhat or much worse.

**Figure 3:**
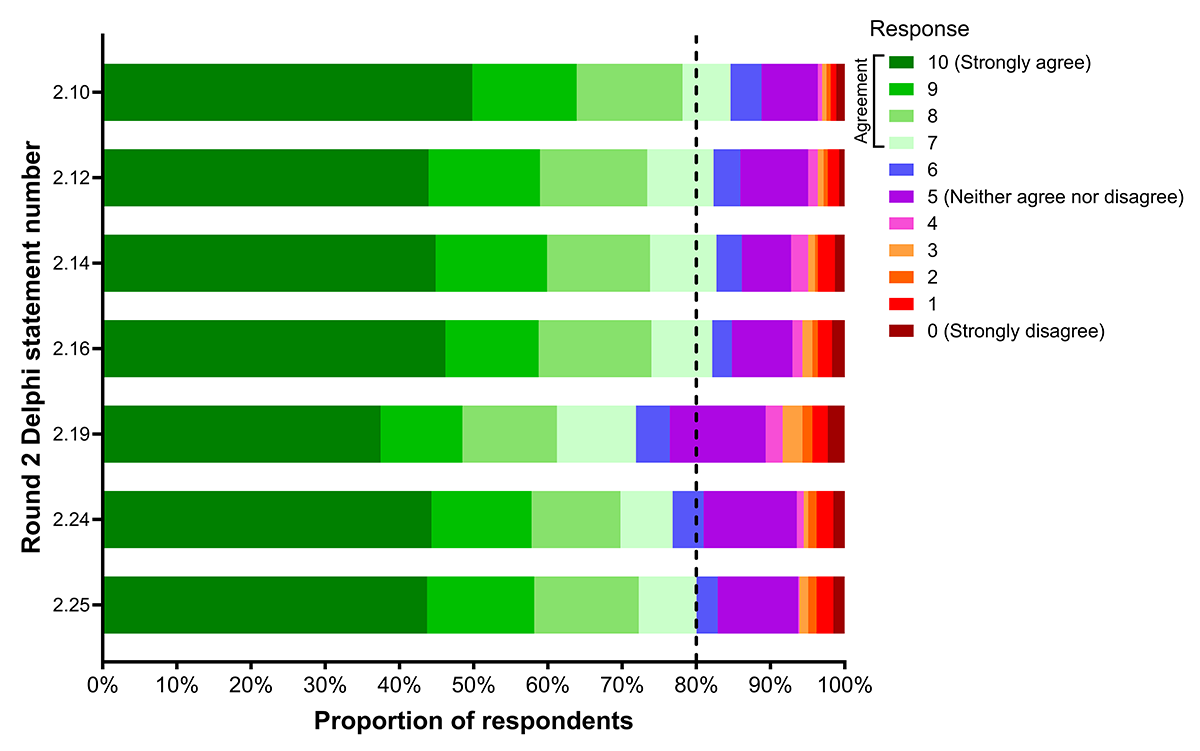
Representation of participants’ level of agreement (rated on an 11-point Likert scale; 0-10) with each of 7 Round 2 Delphi statements (n=526). Participants with a response ≥7 were considered to have agreed with the statement. Statements with strong agreement (≥80%; black dotted line) were accepted.

Figure 3 and Table 4 summarize participant responses to the Round 2 Delphi statements. Respondents strongly agreed that they would like access to instructions on how to test their own physical function and monitor changes (Statement 2.10; 85% agreement) and that having access to information on physical function would allow them to have more informed conversations with health care professionals (Statement 2.12; 82% agreement). Respondents also strongly agreed they would be willing to participate in a remote test of their physical function (Statement 2.14; 83% agreement) and to participate in a remote exercise program suited to their preferences at the time (Statement 2.16; 82% agreement). However, there was only moderate agreement that respondents would be willing to participate in remote exercise programs with a group or individually (Statement 2.19; 72% agreement) and that they would be more likely to participate in a remote physical function test if they were confident that the test was safe and accurate to perform alone (Statement 2.24; 77% agreement). Finally, there was strong agreement that respondents would be more likely to participate in a remote exercise program if they had access to necessary information and resources, including technology and exercise equipment (Statement 2.25; 80% agreement).

**Table 4:** List of Round 2 Delphi statements with levels of agreement and outcome decisions.

Statement Number	Statement	Agreement (%)^a^	Agreement Rating^b^	Outcome
2.10	I would like access to simple and reliable instructions on how to test my physical function myself so that I can monitor how it changes over time	84.6	Strong agreement	Accept
2.12	If I felt I needed help to improve or maintain my physical function, having access to simple information about this (including advice on appropriate health professionals to discuss it with) would help me to have more informed conversations with health professionals about my physical function	82.3	Strong agreement	Accept
2.14	If I felt I needed help to improve or maintain my physical function, I would be willing to participate in a remote test (eg, supervised on a live video call with a health professional or unsupervised using printed instructions and/or video demonstrations provided to me)	82.7	Strong agreement	Accept
2.16	If I felt I needed help to improve or maintain my physical function, I would be willing to participate in a remote exercise program suited to my preferences at the time which may include exercise supervised by a health professional, and/or exercise led by myself	82.1	Strong agreement	Accept
2.19	If I felt I needed help to maintain or improve my physical function, I would be willing to participate in a remote exercise program suited to my preferences at the time which may include exercise performed by myself, and/or exercise performed with a group of people	71.9	Moderate agreement	Reject
2.24	I would be more likely to participate in a remote test of physical function if I was confident that the test was safe and accurate to perform by myself, and I had access to the necessary information and resources, including technology and equipment, to perform the test myself	76.8	Moderate agreement	Reject
2.25	I would be more likely to participate in a remote exercise program if I was confident that I had access to the necessary information and resources, including technology and exercise equipment, to exercise safely and effectively	80.0	Strong agreement	Accept

^a^Proportion of participants who rated statement ≥7 out of 10.

^b^Statement classification based on the following criteria: Strong agreement (>80% of respondents rated statement ≥7 out of 10); Moderate agreement (70% to 80% of respondents rated statement ≥7 out of 10); Low agreement (<70% of respondents rated statement ≥7 out of 10).

### Summary of Delphi Outcomes and Recommendations

Five of seven Round 2 statements achieved consensus (ie, ≥80% agreement), and 2 (2.19 and 2.24) were rejected with moderate agreement. Thus, including the 13 statements accepted in Round 1, a total of 18 statements achieved consensus in this Delphi process and provided a basis for the 7 recommendations relevant to health care professionals, researchers, and policymakers developed by the RAMP Working Group and presented in Table 5.

**Table 5:** Recommendations for remote assessment and management of physical function in older adults.

Recommendation number	Recommendation	Supporting Delphi statements
1	Recognize that physical function is an important health priority for older adults	1.01, 1.02, 1.03, 1.04, 1.05, 1.06
2	Recognize that older adults are aware that it is possible to prevent, delay, and reverse declines in physical function	1.07, 1.08
3	Regularly engage older adults in discussions about their physical function	1.09, 2.12
4	Provide older adults with accessible and reliable information on how to monitor and maintain their physical function	1.11, 1.13, 2.10
5	For older adults who have concerns about their physical function, facilitate physical function assessments in-person and/or remotely considering feasibility and consumer preferences	2.14
6	Facilitate in-person and/or remote exercise programs for improving older adults’ physical function considering feasibility, consumer preferences, and supervision requirements	2.16
7	Ensure that older adults participating in remote assessments or exercise for physical function have access to, or are provided with, appropriate support, technology, and/or equipment to perform assessments and exercises safely and effectively	1.15, 1.21, 2.25

## Discussion

### Principal Results

The World Health Organization’s global strategy on digital health stresses the importance of person-centered approaches where end users are engaged in the design and development phases of digital health approaches [25]. This consumer-focused Delphi process demonstrated that older adults recognize physical function as a health priority and are generally accepting of remote assessment and management, including the use of digital health approaches. Based on these findings, the RAMP Working Group has developed 7 recommendations for researchers, health care professionals, and policymakers to guide remote assessment and management of physical function.

### Comparison with Prior Work

Consistent with previous health care professional surveys showing that assessment and management of physical function are infrequently performed in clinical settings [7], our Round 1 survey demonstrated less than half of older adult respondents had received any health care professional-led assessment or intervention for physical function. Even fewer had participated in a remote physical function assessment (<5%) or intervention (<20%). This is despite almost 60% of respondents having participated in some form of remote care, likely due to increased general digital health use during and since the COVID-19 pandemic [11]. For those respondents who had participated in remote physical function care, several methods were reported, likely influenced by their appropriateness for the desired outcome. For example, video calls were most commonly used for remote physical function assessments whereas written documents were most commonly used for remote interventions. Respondents reported receptiveness to a range of different remote care approaches, and less than 5% stated they would not be willing to participate in any remote care for physical function. Key facilitators for remote care included convenience and flexibility in scheduling, while barriers included lack of guidance and motivation as reported previously [26]. Based on the Delphi statements that achieved consensus, seven recommendations for remote care of physical function in older adults were developed. The above barriers and facilitators should be considered when seeking to implement these recommendations in research, clinical care, and policy. Our findings are consistent with a recent position statement on telehealth policy for older adults, which highlights the need for dedicated policies to address common barriers to telehealth among older adults [27].

### Recommendations for Researchers, Health Care Professionals, and Policymakers

#### Recognize That Physical Function Is an Important Health Priority for Older Adults

Over 97% of respondents strongly agreed physical function was integral to their overall quality of life and their ability to participate in activities with family and friends and in the community, to exercise, and to complete self-care tasks. This recommendation is in line with the World Report on Ageing and Health [28], which emphasizes that maximizing functional ability is a priority for older adults.

#### Recognize That Older Adults are Aware it Is Possible to Prevent, Delay, and Reverse Declines in Physical Function

Over 86% of respondents strongly agreed that it is possible to slow down or prevent poor physical function and that if someone already has poor physical function, it is possible to improve it. This knowledge may encourage older adults to engage in physical function care [29] and health care professionals should leverage it to promote and implement individually tailored strategies to maintain or improve physical function.

#### Regularly Engage Older Adults in Discussions About Their Physical Function

Respondents strongly agreed that they would raise concerns about their physical function with health care professionals and that access to information about physical function would help them initiate these conversations. Previous research has highlighted the importance that older adults place on mutual goal setting with health care professionals regarding their physical function [29], and that aligning care with patient priorities can lead to better health outcomes [30]. Health care professionals may require upskilling to ensure effective collaboration with consumers on identifying causes and symptoms of poor physical function and promoting benefits and strategies for maintaining physical function [8].

#### Provide Older Adults With Accessible and Reliable Information on How to Monitor and Maintain Their Physical Function

In addition to conversations with health care professionals, respondents reported a desire for information on how to independently monitor and maintain their physical function. Previous research has highlighted the importance of providing physical function information and advice to patients [30]. It is necessary to develop and promote appropriate resources that empower older adults to monitor and maintain their physical function.

#### For Older Adults Who Have Concerns About Their Physical Function, Facilitate Physical Function Assessments In-Person and/or Remotely Considering Feasibility and Consumer Preferences

There is a lack of data on the acceptability and appropriateness of remote physical function assessment [31]. In this study, however, respondents agreed they would be willing to participate in a remote physical function assessment if they were concerned about it. Remote assessment of physical function for older adults can be as reliable as face-to-face assessments [32,33] although further research is required to identify the most appropriate physical function tests, protocols, and communication platforms to support reliable remote physical function assessment, as well as facilitators and barriers to implementation in home and community settings.

#### Facilitate In-Person and/or Remote Exercise Programs for Improving Older Adults’ Physical Function Considering Feasibility, Consumer Preferences, and Supervision Requirements

Round 1 statements regarding remote exercise programs with set levels of supervision (ie, always, sometimes, or not supervised) or a set format (ie, individual or group-based) achieved only low to moderate agreement, reflecting the varied preferences for exercise among older adults [34]. Remotely delivered exercise programs with varying levels of synchronous and asynchronous exercise may be beneficial for community-dwelling older adults who do not have access to exercise facilities or prefer exercising alone and/or at home [35]. Remote programs can support novel and engaging exercise approaches such as integrating exercise into everyday activities [36], “exercise snacking” [12,13] and gamification [37], and may also incorporate other behavioral and educational interventions such as nutrition counseling [38].

Many medical professionals lack knowledge on exercise prescription [39]. Referral of patients to an exercise professional with experience in supporting older adults to exercise via remote care should be a consideration for clinicians who do not feel qualified to prescribe exercise. Upskilling exercise professionals in effectively delivering exercise via remote care and providing access to requisite resources and equipment is also important to build capacity for remote management of physical function.

#### Ensure That Older Adults Participating in Remote Assessments or Exercise for Physical Function Have Access to, or Are Provided With, Appropriate Support, Technology, and/or Equipment

Respondents believed it was safe and feasible for them to perform remote physical function assessments and exercise programs, and contrary to previous research [40], had relatively low agreement (<21%) that privacy was a concern. Nevertheless, ensuring remote care is administered by secure technologies and adheres to privacy laws is an important consideration for health care professionals to reduce barriers for those who do have concerns. Most of our respondents strongly agreed that they were comfortable using technology to participate in remote tests and treatments for their physical function. Technological literacy can be a barrier to participation in remote health assessments [26] and further research is required to identify approaches to overcoming technological barriers to remote care. Our results demonstrate that older adults agree that access to appropriate instructions, technology, and equipment would increase the likelihood of participation in remote programs. These findings suggest that older adults can successfully participate in remotely delivered exercise programs if appropriate support is provided. This can include standby technical assistance and technology orientation sessions, especially in the early stages of the program [41]. This support can potentially be integrated into existing funding models to support remote care which have increased internationally, particularly following the COVID-19 pandemic, although further development of policy and reimbursement mechanisms is needed for sustainable integration [42].

### Recommendations for Future Studies

Based on the current results, it is recommended that future research, informed by collaboration with consumers, caregivers, health care professionals, and policymakers should focus on, but not be limited to, the following outcomes:

- Identifying and addressing barriers to accessibility of remote physical function assessment and management for older populations, particularly in culturally and linguistically diverse populations, those with socioeconomic disadvantage, those from low-middle-income countries, and/or those with low technology literacy or with limited access to technology
- Determining optimal approaches (including protocols and technologies) to delivering remote care for physical function to ensure validity and reliability of assessments and effectiveness and safety of interventions
- Exploring cost-effectiveness and implementation processes to embed remote care for physical function across varying levels of health care internationally
- Developing evidence-based guidelines and health promotion strategies for remote physical function assessment and management in older adults

### Strengths and Limitations

Our modified Delphi study was co-developed with health care consumers and incorporated 2 rounds of iterative and anonymous questionnaires and controlled feedback to create consensus. Our study adhered to quality evaluation metrics for Delphi methodology [22] and included an international population of older adults. A high level of agreement (80%) was set a priori for acceptance of statements. There was low attrition of participants between Delphi rounds (<20%), suggesting the respondents were engaged and interested in sharing their views on physical function assessment and management.

Despite these strengths, there were limitations to our study. Given our survey was electronic, the participants required internet access and were likely technologically savvy, and thus a selection bias may be present. Respondents were from 15 generally high-income countries, with the majority residing in Australia and the United Kingdom, a large proportion (76%) were tertiary-educated, and all were English-speaking. It is not known if results are generalizable to those with lower socioeconomic status and/or non–English-speaking individuals. Our respondents also included a large proportion of women (76%), so it may not accurately reflect the views and experiences of older men. Similarly, less than 5% of respondents were aged 80 years or older, and less than 7% had a SARC-F score ≥4 (symptomatic of poor physical function) [24]. However, comparable SARC-F data in our study (median 1, IQR 0-2, proportion with score ≥4=7%) and other similar cohorts (median 0, IQR 1-2, proportion with score ≥4=6%-15%) [24] suggests the level of poor physical function in our sample is generally representative of community-dwelling older adults. Overall, further research investigating remote physical function care preferences in more diverse populations is required to understand the unique and common barriers, enablers, and needs, which would help to enable more widespread adoption of remote methods. This should include research in men, non–English speakers, the oldest old, those with poorer health and/or digital literacy, those with poorer access to health care and/or technology, and those with poor physical function. Furthermore, effective strategies to address technology access, digital literacy, and support needs for these vulnerable populations need to be explored. This may include reducing access barriers through device and internet connectivity provision, improving digital health literacy through ongoing human coaching and troubleshooting support, and co-designing content, interfaces, and delivery models with underserved communities [43].

The current study captures respondents’ preferences and intentions, but not their behaviors or health outcomes. Future research is therefore needed to evaluate engagement with remote physical function interventions among older adults in the real world, as well as the effectiveness of such interventions for relevant health outcomes. The study was also focused on consumer perspectives and does not capture the perspectives of other stakeholders involved in delivering care to older adults (eg, health care professionals and policymakers), which are critical to translating research into practice. To address this, the RAMP working group has recently completed a Delphi process investigating the views of experts involved in the care of older adults (manuscript under review).

### Conclusions

This international consumer Delphi process achieved consensus on 18 Delphi statements, which were synthesized into 7 recommendations for health care professionals, researchers, and policymakers to inform remote assessment and management of physical function in older adults. Further research on the feasibility and integration of remote delivery of physical function assessment and exercise programs is required, and this should be co-designed with older adults and other relevant stakeholders. Furthermore, given the recommendations reflect the sample of predominantly highly educated and digitally literate volunteers from high-income countries, further research is also required to explore their generalizability to more diverse older adult populations.

## Data Availability

The study data may be made available upon reasonable request to the corresponding author.

## Appendix

Multimedia Appendix 1 Study design of the RAMP Consumer Delphi Process.

Multimedia Appendix 2 RAMP Delphi Survey: Round 1. RAMP: Remote Assessment and Management of Physical Function in Older Adults.

Multimedia Appendix 3 RAMP Round 1 results as presented to consumers. RAMP: Remote Assessment and Management of Physical Function in Older Adults.

Multimedia Appendix 4 Summary of changes to Round 1 Delphi statements with moderate or low agreement.

Multimedia Appendix 5 RAMP Delphi Survey: Round 2. RAMP: Remote Assessment and Management of Physical Function in Older Adults.

Multimedia Appendix 6 SARC-F scores for RAMP participants in Round 1. RAMP: Remote Assessment and Management of Physical Function in Older Adults.

Multimedia Appendix 7 Delivery preferences and perceived positives and negatives of remote tests and treatments for physical function among RAMP participants in Round 1. RAMP: Remote Assessment and Management of Physical Function in Older Adults.

## Abbreviations

RAMP: Remote Assessment and Management of Physical Function in Older Adults
