# Supplementary figures and images for: Developing Consumer Consensus on Remote Assessment and Management of Physical Function in Older Adults (RAMP): International Modified Delphi Process
Source: JMIR Aging. 2026 Feb 6;9:e75791. doi: 10.2196/75791 (PMC12924037; doi:10.2196/75791)

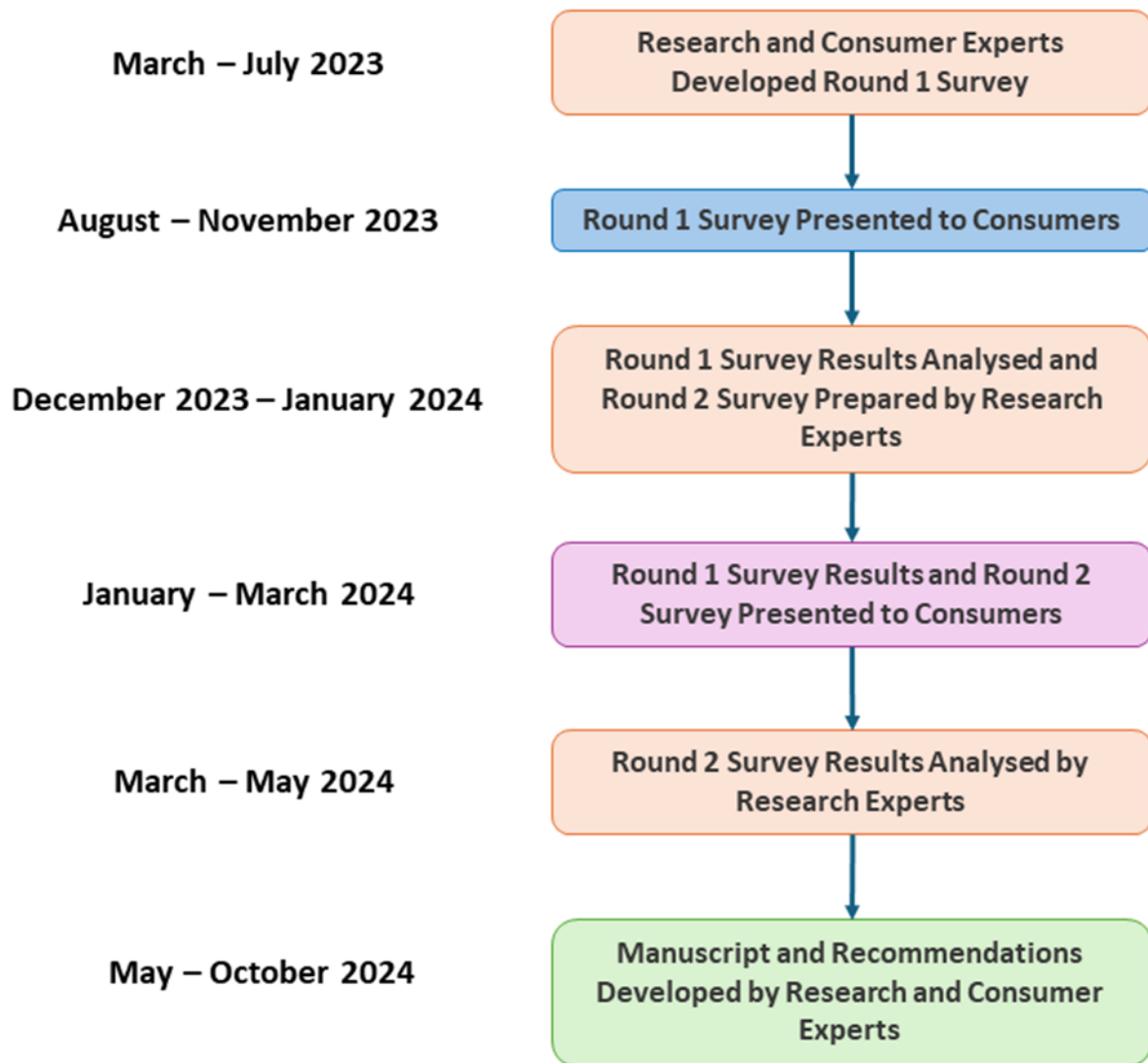

**Multimedia Appendix 1.** Study design of the RAMP Consumer Delphi Process.

Supplement: Multimedia Appendix 1 [file aging_v9i1e75791_app1.pdf]

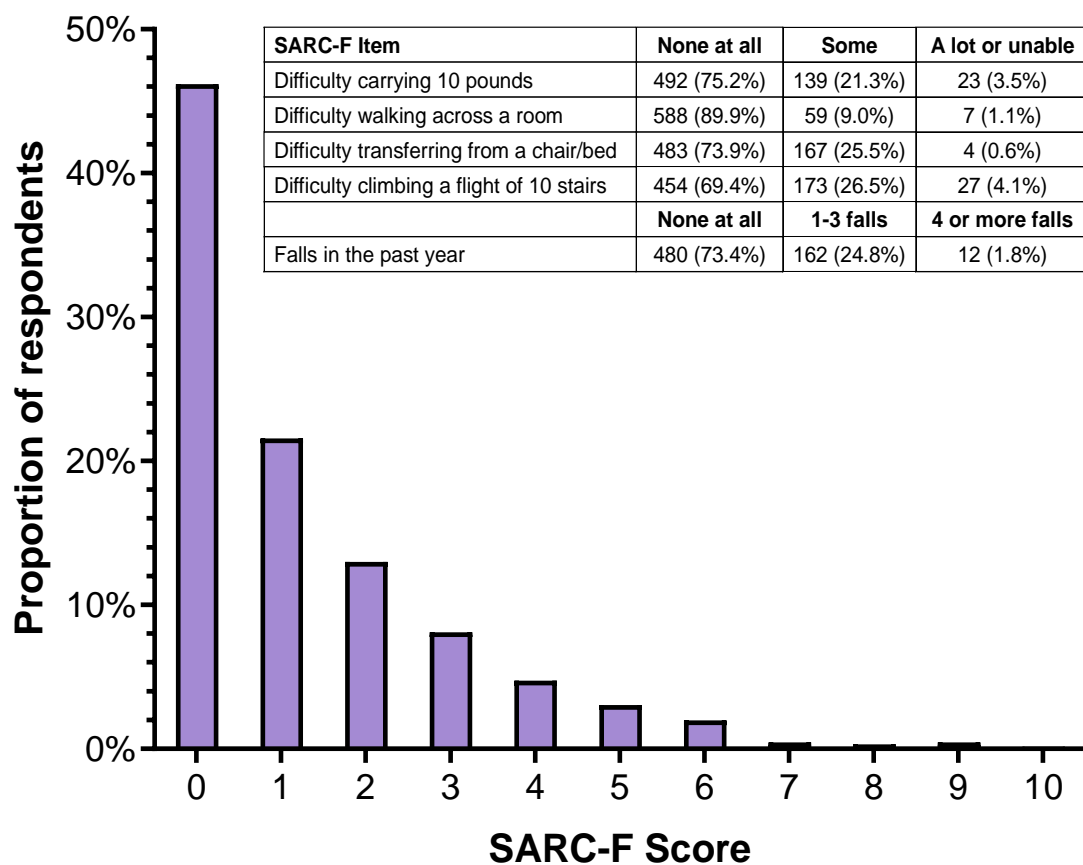

**Multimedia Appendix 6.** SARC-F scores for RAMP participants in Round 1.

Supplement: Multimedia Appendix 6 [file aging_v9i1e75791_app6.pdf]
