# Supplementary material for: Developing Consumer Consensus on Remote Assessment and Management of Physical Function in Older Adults (RAMP): International Modified Delphi Process
Source: JMIR Aging. 2026 Feb 6;9:e75791. doi: 10.2196/75791 (PMC12924037; doi:10.2196/75791)
Supplement: Multimedia Appendix 2 [file aging_v9i1e75791_app2.pdf]

RAMP Delphi Survey 1

Start of Block: Plain Language Summary

Q1.1

Thank you for contributing to this important study on physical function in older adults.

What is physical function?
'Physical function' is the ability of a person to perform everyday activities. Such activities may include walking, climbing stairs, carrying shopping bags, preparing meals, and cleaning your house. Having good physical function allows people to perform everyday activities so that they can stay independent and enjoy a good quality of life.

Having **poor physical function** can limit a person's ability to move about their home and community, and participate in work, hobbies, and time with family and friends. Poor physical function can be caused by factors including low physical activity (e.g. bed rest), chronic diseases (e.g. heart disease), illnesses (e.g. flu), or injuries (e.g. a breaking a bone).

However, the group most commonly affected by poor physical function is adults aged 60 years or older. This is because as we get older, our muscles get weaker, making it more difficult to move around and do tasks that require muscle strength.

Unfortunately, poor physical function is not regularly diagnosed or treated by health professionals (e.g. doctors, nurses, physiotherapists, exercise physiologists etc). Many people are also unaware that there are things they can do themselves to improve their own physical function.

What is this study about?
The aim of this study is to understand how important physical function is to people aged 60 years and older. We also hope to understand how people would like to receive support from health professionals to address concerns about their poor physical function. Finally, we would like to understand what aspects of poor physical function older adults might be able to address themselves.

Please click the right arrow below to continue the survey. You can also use the left arrow at any time to go back to a previous page of the survey.

Page Break

Q50

This study uses a method known as a "Delphi process". This involves two separate surveys aimed at achieving agreement among participants on important issues related to physical function.

Your participation in this survey, and a shorter follow-up survey, will greatly assist in developing recommendations for. All viewpoints are valued and it is important that you complete both surveys (the second survey will be emailed to you approximately 8 weeks after the current survey closes). Your participation is voluntary and anonymous, and if you have any questions or concerns, please email the principal researcher Associate Professor David Scott (d.scott@deakin.edu.au).

Completing the survey
In this online survey, you will be asked to respond to a range of questions and statements. The survey should take less than 15 minutes to complete.

You will be asked questions about yourself and your opinions about physical function. For some questions, you will be asked to choose from a range of responses. For others, you will be asked to rate your level of agreement with a given statement. You can add comments to your responses to provide us with more information if you wish to do so.

End of Block: Plain Language Summary

Start of Block: Eligibility

*

Q2.1 **Please enter your email address below.** We will only use your email address to contact you with information related to this study, and will not share this with anyone outside of the research team.${Q2.1/ChoiceTextEntryValue}

____

*

Q140 **Please confirm your email address below.**

____

Page Break

Q57 Please select your **current age (years)** from the drop-down list below (note only people aged 60 years or older can participate in this study):

▼ 60 (1) ... 120 (61)

Q56 Please click the "I'm not a robot" box below, followed by the right arrow.

End of Block: Eligibility

Start of Block: Demographics Questions

Q3.3 **What is your gender?**

- Male (1)
- Female (2)
- Non-binary / third gender (3)
- Prefer not to say (4)

X→

Q4.1 **Please select the *country you currently live in* from the drop-down list below:**

▼ Afghanistan (1) ... Zimbabwe (1357)

X→

Q52 **Please select the *country you were born in* from the drop-down list below:**

▼ Afghanistan (1) ... Zimbabwe (1357)

Q3.6 **What best describes the highest level of education you have *completed*?**

- ○ No Schooling (1)
- ○ Pre-school (2)
- ○ Infants/Primary School (3)
- ○ Secondary/High School (4)
- ○ University/College or Other Higher Education (5)

Q3.7 **What best describes your *current* employment status?**

- ○ Employed/self-employed full-time (1)
- ○ Employed/self-employed part-time (2)
- ○ Unemployed (3)
- ○ Retired (4)
- ○ Student (5)
- ○ Home Duties (6)
- ○ Pension (including disability or sole-parent pension) (7)

Q3.8 **Would you say that in general your health is (please tick):**

- ◯ Excellent (1)
- ◯ Very Good (2)
- ◯ Good (3)
- ◯ Fair (4)
- ◯ Poor (5)

End of Block: Demographics Questions

Start of Block: Current Physical Function and Experiences Questions

Q8.1 **Compared with when you were 40 years old, would you say your physical function is *currently*:**

- ◯ Much worse (1)
- ◯ Somewhat worse (2)
- ◯ Neither better nor worse (3)
- ◯ Somewhat better (4)
- ◯ Much better (5)

Q5.1 **How much difficulty do you have in lifting and carrying 10 pounds / 4.5 kilograms (e.g. about the weight of an ironing board or two normal-sized house bricks)?**

- ◯ None at all (1)
- ◯ Some (2)
- ◯ A lot or unable (3)

Q5.2 **How much difficulty do you have walking across a room?**

- None at all (1)
- Some (2)
- A lot, use walking aids (e.g. walking frame or walking stick), or unable (3)

Q5.3 **How much difficulty do you have transferring (i.e., getting up) from a chair or bed?**

- None at all (1)
- Some (2)
- A lot or unable (3)

Q5.4 **How much difficulty do you have climbing a flight of 10 stairs?**

- None at all (1)
- Some (2)
- A lot or unable (3)

Q5.5 **How many times have you fallen in the past year?**

- None at all (1)
- 1 - 3 falls (2)
- 4 or more falls (3)

Page Break

Q8.6 **In the past five years, have you started a conversation with a health professional (e.g., doctor, physiotherapist, nurse etc) about your physical function (e.g., asking how you can continue to stay independent as you get older, or why you might not be as strong as you were when you were younger)?**

- ○ Yes (1)
- ○ No (2)
- ○ Don't know (3)

Q58 You may provide further details if you wish:

____

Q59 **In the past five years, has a health professional (e.g., doctor, physiotherapist, nurse etc) started a conversation with you about your physical function?**

- ○ Yes (1)
- ○ No (2)
- ○ Don't know (3)

Q60 You may provide further details if you wish:

____

Q6.2 **Have you ever tried to find information about physical function from sources other than a health professional (e.g., by asking a friend or family member, visiting a website, or reading a book or magazine)?**

○ Yes (1)

○ No (2)

○ Don't know (3)

Q61 You may provide further details if you wish:

____

Q6.3 **Have you ever completed a physical function test (e.g., walking speed test, hand grip strength test, chair stand test) under the supervision of a health professional (e.g., where a health professional asked you to perform a specific test while under their supervision to determine whether your physical function was poor)?**

○ Yes (1)

○ No (2)

○ Don't know (3)

Q62 You may provide further details if you wish:

____

Q6.4 **Have you ever completed a physical function test (e.g., walking speed test, hand grip strength test, chair stand test) while NOT under the supervision of a health**

professional (e.g., performing a specific test designed to determine whether your physical function is poor after you read or viewed instructions in a document or online)?

- ◯ Yes (1)
- ◯ No (2)
- ◯ Don't know (3)

Q63 You may provide further details if you wish:

____

Q65 **Has a health professional ever prescribed you an exercise program aimed at improving your physical function?**

- ◯ Yes (1)
- ◯ No (2)
- ◯ Don't know (3)

Q66 You may provide further details if you wish:

____

Q67 **Have you ever commenced an exercise program aimed at improving your physical function while NOT under the supervision of a health professional (e.g., an exercise**

program that you created for yourself, with or without the help of a friend or family member, or using a website/book/magazine etc)?

- Yes (1)
- No (2)
- Don't know (3)

Q68 You may provide further details if you wish:

____

End of Block: Current Physical Function and Experiences Questions

---

Start of Block: Physical Function Statements

Q8.2 **Please rate your level of agreement with the following statements on physical function, where: *0 = strongly disagree, 5 = neither agree nor disagree, 10 = strongly agree.* To respond to each statement, *drag the slider* to the number that best corresponds with your level of agreement/disagreement.**

Having good physical function is important to the overall quality of life of older adults:

	0 1 2 3 4 5 6 7 8 9 10
0 = Strongly disagree; 5 = neither agree nor disagree; 10 = Strongly agree ()	

Q80 You may provide further details if you wish:

____

Q81 **Having good physical function is important for activities involving moving around the community (e.g. going shopping or to a restaurant or cafe, visiting your neighbours, friends and family or the doctor etc):**

	0	1	2	3	4	5	6	7	8	9	10
0 = Strongly disagree; 5 = neither agree nor disagree; 10 = Strongly agree ()											

Q82 You may provide further details if you wish:

____

Q83 **Having good physical function is important for participating in activities with family and friends (e.g. playing with grandchildren):**

	0	1	2	3	4	5	6	7	8	9	10
0 = Strongly disagree; 5 = neither agree nor disagree; 10 = Strongly agree ()											

Q84 You may provide further details if you wish:

____

Q85 **Having good physical function is important for participating in activities like work, household duties (e.g. cooking, cleaning, gardening), and volunteering:**

0 1 2 3 4 5 6 7 8 9 10

0 = Strongly disagree; 5 = neither agree nor disagree; 10 = Strongly agree ()	

Q86 You may provide further details if you wish:

____

Q85b **Having good physical function is important for participating in hygiene activities (e.g. showering, dressing, using the toilet):**

	0	1	2	3	4	5	6	7	8	9	10
0 = Strongly disagree; 5 = neither agree nor disagree; 10 = Strongly agree ()											

Q85b.2 You may provide further details if you wish:

____

Q87 **Having good physical function is important for participating in exercise (e.g. walking, swimming, dancing, golf and other types of physical activity):**

	0	1	2	3	4	5	6	7	8	9	10
0 = Strongly disagree; 5 = neither agree nor disagree; 10 = Strongly agree ()											

Q88 You may provide further details if you wish:

____

Q91 **It is possible to slow down or prevent poor physical function that occurs as we get older:**

	0	1	2	3	4	5	6	7	8	9	10
0 = Strongly disagree; 5 = neither agree nor disagree; 10 = Strongly agree ()											

Q89 You may provide further details if you wish:

____

Q79 **If someone already has poor physical function, it is possible to improve it:**

	0	1	2	3	4	5	6	7	8	9	10
0 = Strongly disagree; 5 = neither agree nor disagree; 10 = Strongly agree ()											

Q92 You may provide further details if you wish:

____

Q90 **If I was concerned about my physical function, I would discuss it with my health professional:**

	0	1	2	3	4	5	6	7	8	9	10
0 = Strongly disagree; 5 = neither agree nor disagree; 10 = Strongly agree ()											

Q78 You may provide further details if you wish:

____

Q95 **I would like access to information about how to test my physical function myself to determine if it is poor:**

	0 1 2 3 4 5 6 7 8 9 10
0 = Strongly disagree; 5 = neither agree nor disagree; 10 = Strongly agree ()	

Q94 You may provide further details if you wish:

____

Q95b **I would like access to information about things that I can do myself to improve my physical function:**

	0 1 2 3 4 5 6 7 8 9 10
0 = Strongly disagree; 5 = neither agree nor disagree; 10 = Strongly agree ()	

Q95b.2 You may provide further details if you wish:

____

Q96 **Having better access to information on physical function would help me to have conversations about this with health professionals:**

0 1 2 3 4 5 6 7 8 9 10

0 = Strongly disagree; 5 = neither agree nor disagree; 10 = Strongly agree ()	

Q98 You may provide further details if you wish:

____

Q97 **Having better access to information on physical function would help me to take care of my own physical function:**

	0	1	2	3	4	5	6	7	8	9	10
0 = Strongly disagree; 5 = neither agree nor disagree; 10 = Strongly agree ()											

Q99 You may provide further details if you wish:

____

End of Block: Physical Function Statements

Start of Block: Remote Health Care Questions

Q6.5 **The following questions focus on your *remote health care* experiences. *'Remote health care'* refers to health care services where the patient is not in the same location as the health professional.**

This could commonly include a 'telehealth' appointment where a patient connects with their doctor via telephone or video call to discuss a health issue or receive advice on medication etc.

It could also include a patient following a rehabilitation program by themselves at home using printed instructions, or using video demonstrations within a smartphone application or website provided by a physiotherapist or other health professional.

Have you ever participated in any type of *remote health care service*?

○ Yes (1)

○ No (2)

○ Don't know (3)

Q64 You may provide further details if you wish:

____

Display This Question:

If The following questions focus on your remote health care experiences. 'Remote health care' refers... = Yes

Q127 **Which of the below methods have you used to participate in a remote health care service? *(Please select all that apply)*:**

- ☐ Written documents (e.g. flyers, brochures magazines, books etc) (1)
- ☐ Videos (e.g. on a website or DVD) (2)
- ☐ Telephone calls (3)
- ☐ Video calls (e.g. FaceTime, Zoom etc) (4)
- ☐ Websites (5)
- ☐ Emails or text messages (6)
- ☐ Smartphone or tablet applications (7)
- ☐ Other (please specify) (8) ____

Display This Question:

If The following questions focus on your remote health care experiences. 'Remote health care' refers... = Yes

Q128 You may provide further details if you wish:

____

Display This Question:

If The following questions focus on your remote health care experiences. 'Remote health care' refers... = Yes

Q6.6 **Please rate your overall experience of remote health care:**

- ◯ Very positive (1)
- ◯ Somewhat positive (2)
- ◯ Neutral (3)
- ◯ Somewhat negative (4)
- ◯ Very negative (5)

Display This Question:

If The following questions focus on your remote health care experiences. 'Remote health care' refers... = Yes

Q70 You may provide further details if you wish:

____

Q69 **Have you ever participated in a *remote test* of your physical function by a health professional (e.g., where a health professional has asked you to perform a walking test or a similar test on a video call, or in another way where the health professional is in a different location to you)?**

- ◯ Yes (1)
- ◯ No (2)
- ◯ Don't know (3)

Q130 You may provide further details if you wish:

____

Display This Question:

If Have you ever participated in a remote test of your physical function by a health professional (e... = Yes

Q129 **Which of the below methods have you used to participate in a remote test of your physical function? *(Please select all that apply)*:**

- ☐ Written documents (e.g. flyers, brochures magazines, books etc) (1)
- ☐ Videos (e.g. on a website or DVD) (2)
- ☐ Telephone calls (3)
- ☐ Video calls (e.g. FaceTime, Zoom etc) (4)
- ☐ Websites (5)
- ☐ Emails or text messages (6)
- ☐ Smartphone or tablet applications (7)
- ☐ Other (please specify) (8) ____

Display This Question:

If Have you ever participated in a remote test of your physical function by a health professional (e... = Yes

Q71 You may provide further details if you wish:

____

Display This Question:

If Have you ever participated in a remote test of your physical function by a health professional (e... = Yes

Q6.9 **Please rate your overall experience of participating in remote tests of your physical function:**

- ◯ Very positive (1)
- ◯ Somewhat positive (2)
- ◯ Neutral (3)
- ◯ Somewhat negative (4)
- ◯ Very negative (5)

Display This Question:

If Have you ever participated in a remote test of your physical function by a health professional (e... = Yes

Q76 You may provide further details if you wish:

____

Page Break

Q72 **Have you ever participated in *remote treatment* of your physical function by a health professional (e.g., where a health professional has prescribed you an exercise program to perform where the health professional is in a different location to you)?**

- ◯ Yes (1)
- ◯ No (2)
- ◯ Don't know (3)

Q73 You may provide further details if you wish:

____

Display This Question:

If Have you ever participated in remote treatment of your physical function by a health professional... = Yes

Q132 **Which of the below methods have you used to participate in a remote treatment of your physical function? *(Please select all that apply)*:**

- ▢ Written documents (e.g. flyers, brochures magazines, books etc) (1)
- ▢ Videos (e.g. on a website or DVD) (2)
- ▢ Telephone calls (3)
- ▢ Video calls (e.g. FaceTime, Zoom etc) (4)
- ▢ Websites (5)
- ▢ Emails or text messages (6)
- ▢ Smartphone or tablet applications (7)
- ▢ Other (please specify) (8) ____

Display This Question:

If Have you ever participated in remote treatment of your physical function by a health professional... = Yes

Q133 You may provide further details if you wish:

____

Display This Question:

If Have you ever participated in remote treatment of your physical function by a health professional... = Yes

Q75 **Please rate your overall experience of participating in remote treatment of your physical function:**

- ○ Very positive (1)
- ○ Somewhat positive (2)
- ○ Neutral (3)
- ○ Somewhat negative (4)
- ○ Very negative (5)

Display This Question:

If Have you ever participated in remote treatment of your physical function by a health professional... = Yes

Q77 You may provide further details if you wish:

____

End of Block: Remote Health Care Questions

Start of Block: Remote Assessment and Prescription Questions

Q100 **Please rate your level of agreement with the following statements on remote tests and treatment for physical function, where:** ***0 = strongly disagree,*** ***5 = neither agree nor disagree,*** ***10 = strongly agree.***
To respond to each statement, *drag the slider* to the number that best corresponds with your level of agreement/disagreement.

I would be willing to participate in remote tests of my physical function (e.g., on a video

call with a health professional, or by myself using written instructions and/or video demonstrations provided to me):

	0	1	2	3	4	5	6	7	8	9	10
0 = Strongly disagree; 5 = neither agree nor disagree; 10 = Strongly agree ()											

Q101 You may provide further details if you wish:

____

Q104 **I am confident that it would be safe for me to perform physical function tests at home without direct supervision by a health professional if I was provided with instructions (e.g., written information, video demonstrations):**

	0	1	2	3	4	5	6	7	8	9	10
0 = Strongly disagree; 5 = neither agree nor disagree; 10 = Strongly agree ()											

Q103 You may provide further details if you wish:

____

Q105 **I would be willing to participate in a remote exercise program to improve my physical function if it was ALWAYS supervised (e.g., exercising while on a live video call with a health professional for all exercise sessions):**

	0	1	2	3	4	5	6	7	8	9	10
0 = Strongly disagree; 5 = neither agree nor disagree; 10 = Strongly agree ()											

Q108 You may provide further details if you wish:

____

Q106 **I would be willing to participate in a remote exercise program if it was SOMETIMES supervised (e.g., exercising on a live video call with a health professional for some exercise sessions, but exercising by myself unsupervised using instructions provided by the health professional for other sessions):**

	0 1 2 3 4 5 6 7 8 9 10
0 = Strongly disagree; 5 = neither agree nor disagree; 10 = Strongly agree ()	

Q109 You may provide further details if you wish:

____

Q107 **I would be willing to participate in a remote exercise program if it was NOT supervised (e.g., exercising by myself unsupervised using instructions provided by a health professional):**

	0 1 2 3 4 5 6 7 8 9 10
0 = Strongly disagree; 5 = neither agree nor disagree; 10 = Strongly agree ()	

Q110 You may provide further details if you wish:

____

Q115 **If I was to participate in a remote exercise program I would be happy to do so with a group (e.g., exercising by myself at home but while on a video call with other people like**

me who are also exercising at home, with or without the supervision of a health professional):

	0 1 2 3 4 5 6 7 8 9 10
0 = Strongly disagree; 5 = neither agree nor disagree; 10 = Strongly agree ()	

Q112 You may provide further details if you wish:

____

Q113 **If I was to participate in a remote exercise program to improve my physical function, I would be happy to do so alone without other people like me involved in the exercise sessions (e.g., exercising by myself at home with or without supervision by a health professional):**

	0 1 2 3 4 5 6 7 8 9 10
0 = Strongly disagree; 5 = neither agree nor disagree; 10 = Strongly agree ()	

Q114 You may provide further details if you wish:

____

Q111 **I would be comfortable using technology (e.g., computers, smartphones, tablets etc) to participate in remote tests and treatments for my physical function:**

	0 1 2 3 4 5 6 7 8 9 10
0 = Strongly disagree; 5 = neither agree nor disagree; 10 = Strongly agree ()	

Q116 You may provide further details if you wish:

____

Q117 **I would be concerned about the privacy and security of my personal information when participating in remote tests and treatments for physical function using technology (e.g., computer, smartphone, tablet etc):**

	0	1	2	3	4	5	6	7	8	9	10
0 = Strongly disagree; 5 = neither agree nor disagree; 10 = Strongly agree ()											

Q118 You may provide further details if you wish:

____

Q119 **Remote physical function tests or exercise programs would be difficult to perform in my home (e.g., because there is limited space):**

	0	1	2	3	4	5	6	7	8	9	10
0 = Strongly disagree; 5 = neither agree nor disagree; 10 = Strongly agree ()											

Q120 You may provide further details if you wish:

____

Q7.3 **Which of the below methods would you be willing to use in order to participate in remote tests and treatments for physical function? *(Please select all that apply)*:**

▢ Written documents (e.g. flyers, brochures magazines, books etc) (1)

▢ Videos (e.g. on a website or DVD) (2)

▢ Telephone calls (3)

▢ Video calls (e.g. FaceTime, Zoom etc) (4)

▢ Websites (5)

▢ Emails or text messages (6)

▢ Smartphone or tablet applications (7)

▢ Other (please specify) (8) ____

▢ ⊗None of the above (I would not be willing to participate in remote tests or treatments for physical function) (9)

Q121 You may provide further details if you wish:

____

Display This Question:

If Which of the below methods would you be willing to use in order to participate in remote tests an... != None of the above (I would not be willing to participate in remote tests or treatments for physical function)

Q7.4 **In the *initial* stages of a remote exercise program (e.g. first 3 months), how often would you prefer to have check-ins or consultations with a health professional?**

○ Daily (or every time I exercise) (1)

○ Weekly (2)

○ Every second week (3)

○ Monthly (4)

○ As recommended by health professional (6)

Display This Question:

If Which of the below methods would you be willing to use in order to participate in remote tests an... != None of the above (I would not be willing to participate in remote tests or treatments for physical function)

Q138 You may provide further details if you wish:

____

Display This Question:

If Which of the below methods would you be willing to use in order to participate in remote tests an... != None of the above (I would not be willing to participate in remote tests or treatments for physical function)

Q139 ***After* the initial stages of a remote exercise program (e.g. after the first 3 months), do you think you would be willing to reduce the frequency of check-ins or consultations with a health professional?**

○ Yes, I think I would require less support from a health professional after the initial stages (1)

○ No, I think I would require the same amount or more support from a health professional after the initial stages (2)

○ Not sure (3)

Display This Question:

If Which of the below methods would you be willing to use in order to participate in remote tests an... != None of the above (I would not be willing to participate in remote tests or treatments for physical function)

Q134 You may provide further details if you wish:

____

Q7.5 **Which of the below factors do you perceive as potential positives of remote tests and treatments for physical function? *(Select all that apply)*:**

- ▢ Convenience of not needing to travel to appointments with health professionals (1)
- ▢ Ability to access health professionals who would otherwise be difficult to access in-person (e.g. due to living in a rural location with limited health services) (2)
- ▢ Lower cost compared to attending in-person sessions (3)
- ▢ Lower waiting times to see a health professional (7)
- ▢ Flexibility to perform assessments and exercises when convenient (4)
- ▢ Ability to complete tasks at my own pace (5)
- ▢ Other (please specify) (6) ____

Q135 You may provide further details if you wish:

____

Q7.6 **Which of the below factors do you perceive as potential negatives of remote tests and treatments for physical function? *(Select all that apply)*:**

- ☐ Performing tests and exercises without supervision in my home may be unsafe (1)
- ☐ Performing tests and exercises without supervision in my home may not be effective (2)
- ☐ Lack of personalised guidance during exercises (3)
- ☐ Lack of social interaction and motivation from peers or professionals (4)
- ☐ Difficulty using technology (5)
- ☐ Concerns about privacy and security when using technology (6)
- ☐ Other (please specify) (7) ____

Q136 You may provide further details if you wish:

____

Q7.7 **Do you have any other thoughts or concerns about remote assessment and management of your physical function that you would like to share?**

____

Display This Question:

If List of Countries = Australia

And If

How much difficulty do you have in lifting and carrying 10 pounds / 4.5 kilograms (e.g. about the... != None at all

Or How much difficulty do you have walking across a room? != None at all

Or How much difficulty do you have transferring (i.e., getting up) from a chair or bed? != None at all

Or How much difficulty do you have climbing a flight of 10 stairs? != None at all

Or How many times have you fallen in the past year? != None at all

Q8.11 **Based on the information you have provided, you may be eligible to participate in a subsequent study where we will work with older adults to develop resources that can support them to assess and manage their physical function.**

This study involves participation in two Zoom meetings and participants will be reimbursed for their time.

Are you interested in participating in this subsequent study?

- ○ Yes or maybe - please send me further information regarding this study using my provided email address (1)
- ○ No - please do not contact me regarding this study (2)

Q122

You have reached the end of this survey.

What happens next?

Once this survey closes, the research team will begin conducting an analysis of participants' responses and then prepare a summary of the results and a second, shorter survey. These will both be shared with you via your email address approximately 8 weeks after this survey closes.

If you have any questions or comments in the meantime, please contact Associate Professor David Scott (d.scott@deakin.edu.au).

End of Block: Remote Assessment and Prescription Questions
