# Supplementary material for: Developing Consumer Consensus on Remote Assessment and Management of Physical Function in Older Adults (RAMP): International Modified Delphi Process
Source: JMIR Aging. 2026 Feb 6;9:e75791. doi: 10.2196/75791 (PMC12924037; doi:10.2196/75791)
Supplement: Multimedia Appendix 3 [file aging_v9i1e75791_app3.pdf]

RAMP CONSUMER DELPHI STUDY

Survey 1 Results

Who are you?

654 participants with an average age of 69 years (age range 60 - 92 years)

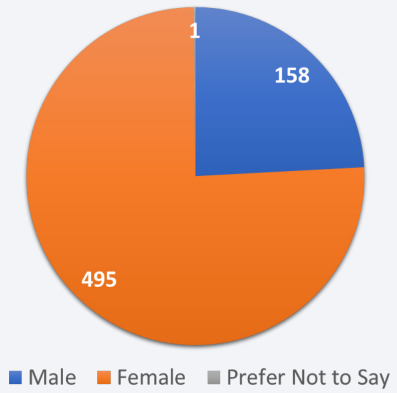

Most participants are female

Participants live in 15 different countries

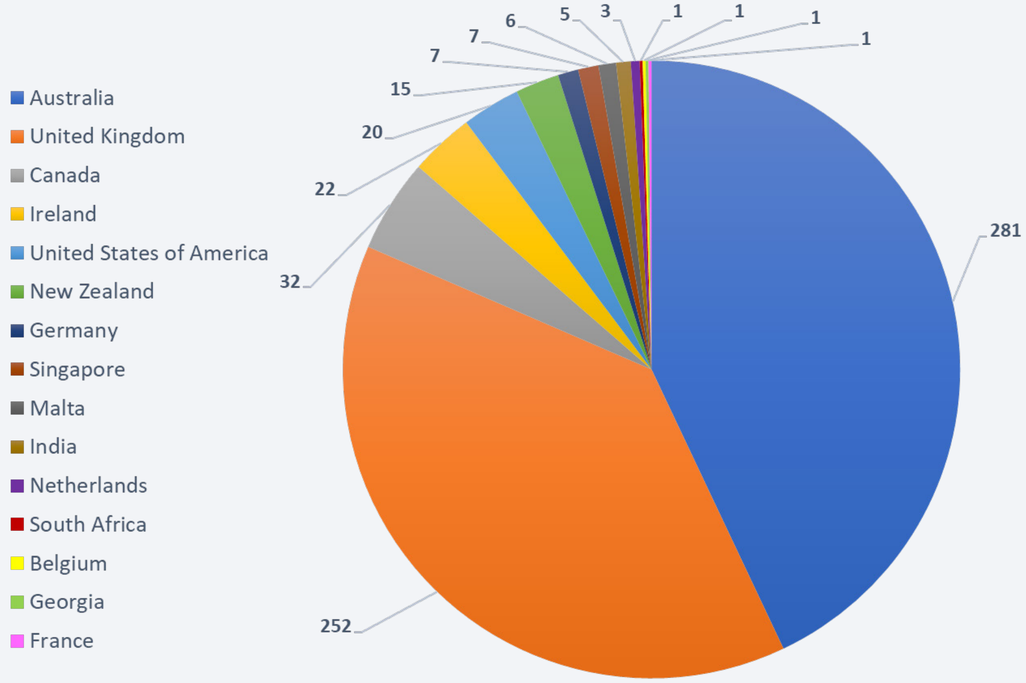

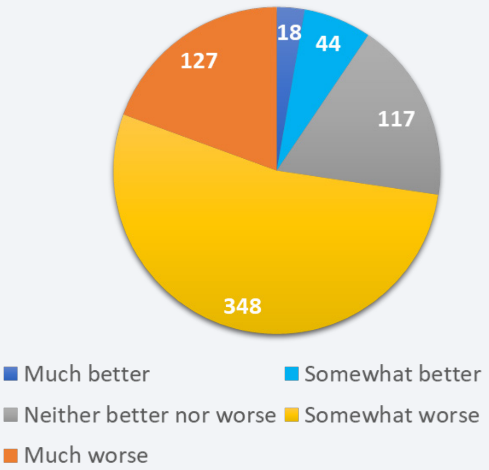

Most participants believe their physical function is worse now than when they were 40 years old

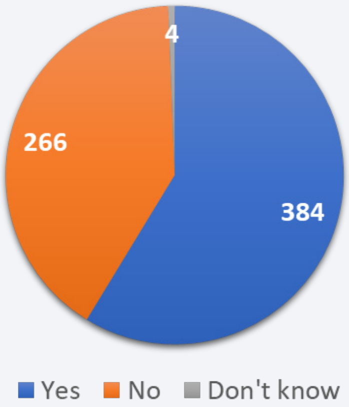

Most participants have received a remote health care service

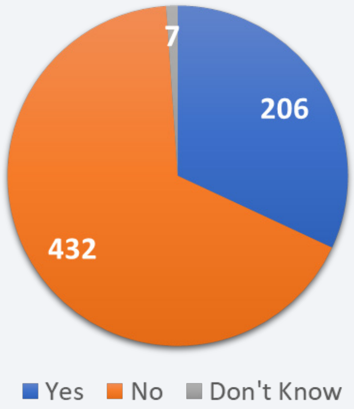

Most participants have not had a discussion about physical function with a health professional in the past 5 years

Most participants have tried to find information about physical function from sources other than a health professional

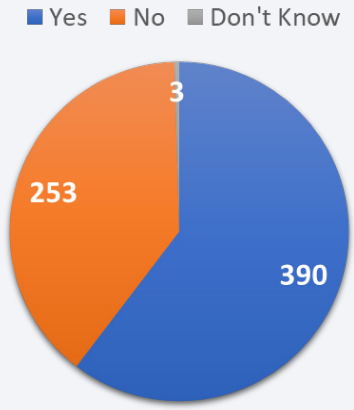

RAMP CONSUMER DELPHI STUDY

Survey 1 Results

Your responses to statements about physical function

The figure below presents the number of participants at each level of agreement (Strongly Agree to Strongly Disagree) for the 23 statements on physical function in Survey 1.

Statements with large amounts of green area generally have strong agreement. These statements are accepted as having achieved consensus and will not be revised for Survey 2.

Statements with greater amounts of orange and grey have low or moderate agreement. These statements have not achieved consensus and will be rejected, or otherwise modified for inclusion in the Survey 2.

You can learn more about the results for each statement and our decisions to accept, reject or modify them on the following pages.

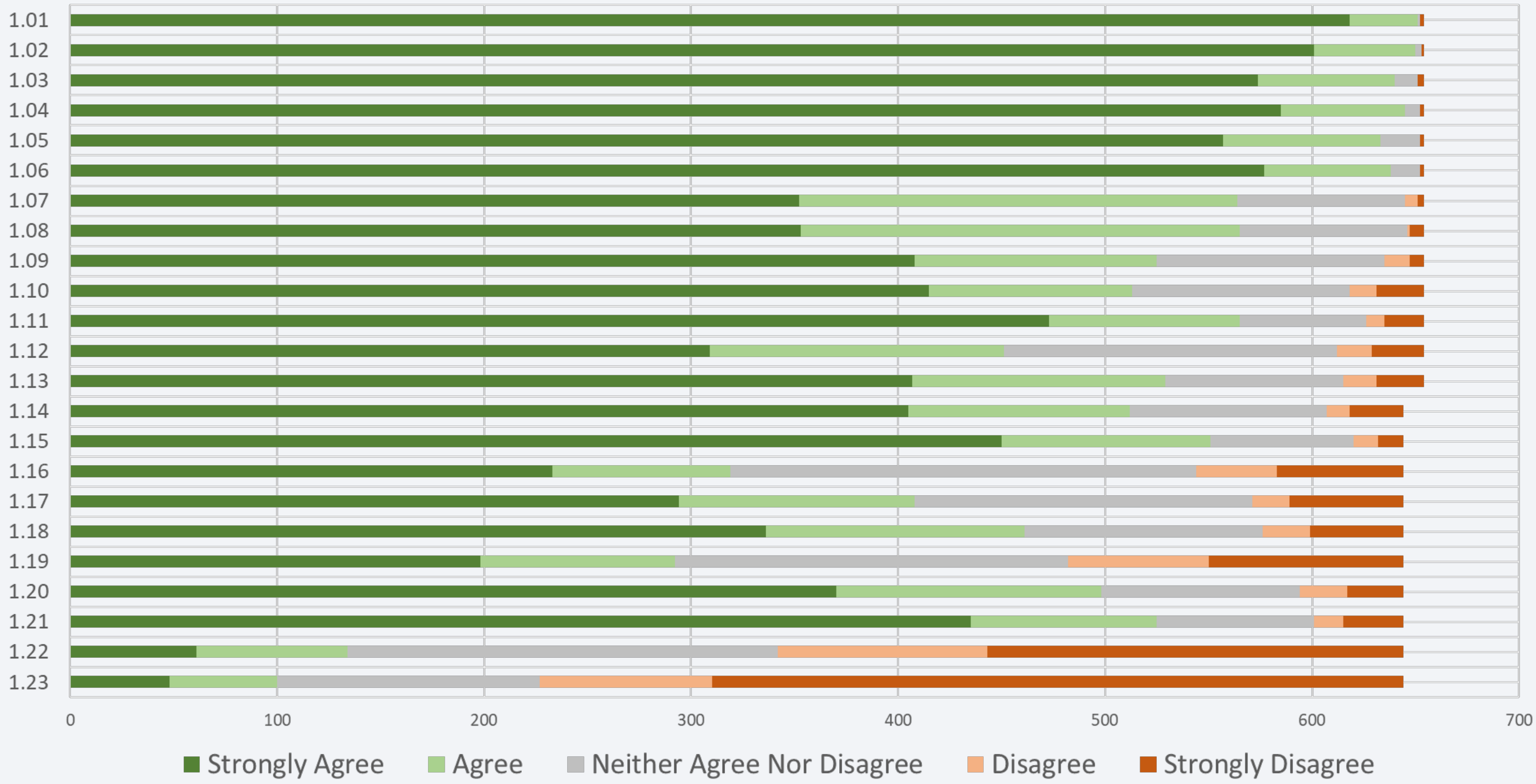

RAMP CONSUMER DELPHI STUDY

Survey 1 Results

Your responses to statements about physical function

Statement Number	Statement	Agreement (%)*	Rating†	Decision
**1.01**	Having good physical function is important to the overall quality of life of older adults	99.54	Strong Agreement	Accept
**1.02**	Having good physical function is important for activities involving moving around the community (e.g., going shopping or to a restaurant or cafe, visiting your neighbours, friends and family or the doctor etc)	99.39	Strong Agreement	Accept
**1.03**	Having good physical function is important for participating in activities with family and friends (e.g., playing with grandchildren)	97.86	Strong Agreement	Accept
**1.04**	Having good physical function is important for participating in activities like work, household duties (e.g., cooking, cleaning, gardening), and volunteering	98.62	Strong Agreement	Accept
**1.05**	Having good physical function is important for participating in hygiene activities (e.g. showering, dressing, using the toilet)	96.79	Strong Agreement	Accept
**1.06**	Having good physical function is important for participating in exercise (e.g., walking, swimming, dancing, golf and other types of physical activity)	97.55	Strong Agreement	Accept
**1.07**	It is possible to slow down or prevent poor physical function that occurs as we get older	86.24	Strong Agreement	Accept
**1.08**	If someone already has poor physical function, it is possible to improve it	86.39	Strong Agreement	Accept
**1.09**	If I was concerned about my physical function, I would discuss it with my health professional	80.28	Strong Agreement	Accept

***Proportion of participants who rated statement ≥7 out of 10**

†Strong agreement (>80% of respondents rated statement ≥7 out of 10)

Moderate agreement (70% to 80% of respondents rated statement ≥7 out of 10)

Low agreement (<70% of respondents rated statement ≥7 out of 10)

RAMP CONSUMER DELPHI STUDY

Survey 1 Results

Your responses to statements about physical function

Statement Number	Statement	Agreement (%)*	Rating†	Decision
**1.10**	I would like access to information about how to test my physical function myself to determine if it is poor	78.44	Moderate Agreement	Revise for Survey 2
**1.11**	I would like access to information about things that I can do myself to improve my physical function	86.39	Strong Agreement	Accept
**1.12**	Having better access to information on physical function would help me to have conversations about this with health professionals	68.96	Low Agreement	Revise for Survey 2
**1.13**	Having better access to information on physical function would help me to take care of my own physical function	80.89	Strong Agreement	Accept
**1.14**	I would be willing to participate in remote tests of my physical function (e.g., on a video call with a health professional, or by myself using written instructions and/or video demonstrations provided to me)	79.5	Moderate Agreement	Revise for Survey 2
**1.15**	I am confident that it would be safe for me to perform physical function tests at home without direct supervision by a health professional if I was provided with instructions (e.g., written information, video demonstrations)	85.56	Strong Agreement	Accept
**1.16**	I would be willing to participate in a remote exercise program to improve my physical function if it was ALWAYS supervised (e.g., exercising while on a live video call with a health professional for all exercise sessions)	49.53	Low Agreement	Revise for Survey 2

***Proportion of participants who rated statement ≥7 out of 10**

†Strong agreement (>80% of respondents rated statement ≥7 out of 10)

Moderate agreement (70% to 80% of respondents rated statement ≥7 out of 10)

Low agreement (<70% of respondents rated statement ≥7 out of 10)

RAMP CONSUMER DELPHI STUDY

Survey 1 Results

Your responses to statements about physical function

Statement Number	Statement	Agreement (%)*	Rating†	Decision
**1.17**	I would be willing to participate in a remote exercise program if it was SOMETIMES supervised (e.g., exercising on a live video call with a health professional for some exercise sessions, but exercising by myself unsupervised using instructions provided by the health professional for other sessions)	63.35	Low Agreement	Revise for Survey 2
**1.18**	I would be willing to participate in a remote exercise program if it was NOT supervised (e.g., exercising by myself unsupervised using instructions provided by a health professional)	71.58	Moderate Agreement	Revise for Survey 2
**1.19**	If I was to participate in a remote exercise program I would be happy to do so with a group (e.g., exercising by myself at home but while on a video call with other people like me who are also exercising at home, with or without the supervision of a health professional)	45.34	Low Agreement	Revise for Survey 2
**1.20**	If I was to participate in a remote exercise program to improve my physical function, I would be happy to do so alone without other people like me involved in the exercise sessions (e.g., exercising by myself at home with or without supervision by a health professional)	77.33	Moderate Agreement	Revise for Survey 2
**1.21**	I would be comfortable using technology (e.g., computers, smartphones, tablets etc) to participate in remote tests and treatments for my physical function	81.52	Strong Agreement	Accept
**1.22**	I would be concerned about the privacy and security of my personal information when participating in remote tests and treatments for physical function using technology (e.g., computer, smartphone, tablet etc)	20.81	Low Agreement	Reject
**1.23**	Remote physical function tests or exercise programs would be difficult to perform in my home (e.g., because there is limited space)	15.53	Low Agreement	Reject

***Proportion of participants who rated statement ≥7 out of 10**

†Strong agreement (>80% of respondents rated statement ≥7 out of 10)

Moderate agreement (70% to 80% of respondents rated statement ≥7 out of 10)

Low agreement (<70% of respondents rated statement ≥7 out of 10)

RAMP CONSUMER DELPHI STUDY

Survey 2 Introduction

How have we modified statements?

Of 23 statements presented to participants in Survey 1, 13 were accepted with strong agreement. Two statements were also rejected with very low agreement. There is no need to re-visit these statements in Survey 2.

The remaining 8 statements were reviewed to understand the reasons for moderate or low agreement. These statements have now been modified and will be presented to you in Survey 2 to assess your level of agreement.

The following pages provide a summary of the decisions we have made in modifying these statements and are presented as follows:

Original statement/s and level of agreement

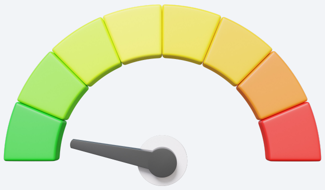

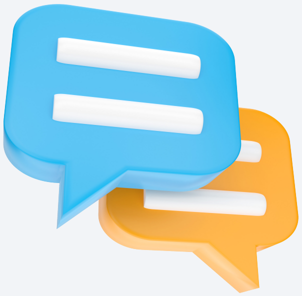

Key reasons for low agreement based on participants' comments

Modified statement/s for Survey 2 with summary of reasons for changes

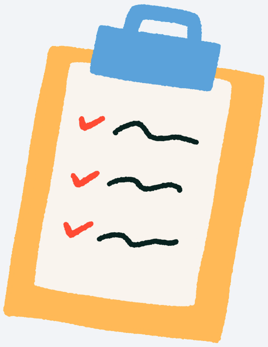

Survey 2 Introduction

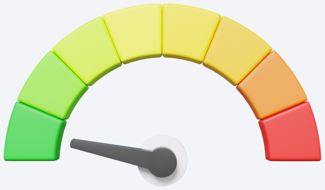

Statement 1.10: *I would like access to information about how to test my physical function myself to determine if it is poor*

<u>Agreement: 78.4%</u>

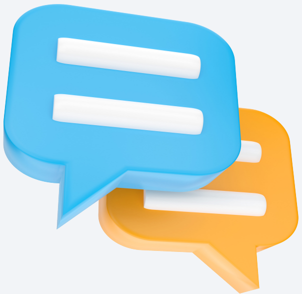

The wording of this statement appears to have been inappropriate as it suggests people may only wish to assess their physical function if they are worried it is poor. Participant comments suggest that some would like to be able to assess their physical function even when it is good, in order to monitor how it changes over time. Other comments highlighted the importance of the information being reliable and easy to follow.

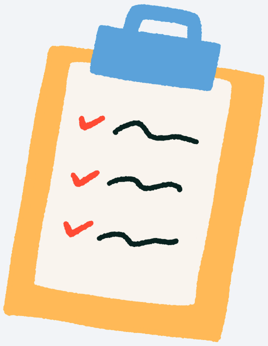

Statement 2.10: *I would like access to simple and reliable instructions on how to test my physical function myself so that I can monitor how it changes over time*

This revised statement recognises the importance of having access to easy to follow information and that people may like to monitor their physical function changes over time.

Statement 2.10 will be presented to participants in Survey 2.

RAMP CONSUMER DELPHI STUDY

Survey 2 Introduction

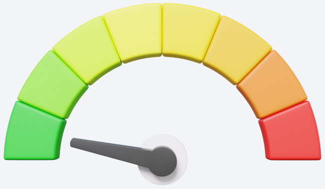

Statement 1.12: *Having better access to information on physical function would help me to have conversations about this with health professionals*

<u>Agreement: 69.0%</u>

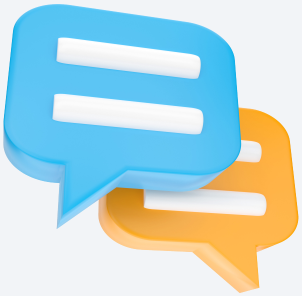

Low agreement for this statement appeared to be influenced by a number of factors including the view that access to health professionals with relevant expertise is limited or, conversely, that participants are already able to have these discussions with their health professional. A common theme was that family doctors/general practitioners do not have time or are not interested in physical function. Those who felt their current physical function levels are already adequate, or that their existing knowledge was adequate, also tended to disagree.

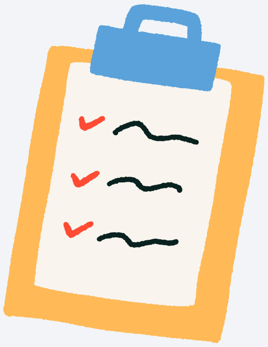

Statement 2.12: *If I felt I needed help to improve or maintain my physical function, having access to simple information about this (including advice on appropriate health professionals to discuss it with) would help me to have more informed conversations with health professionals about my physical function*

This revised statement recognises that discussions about physical function require access to an appropriate health professional and are most likely to occur when people are concerned about their physical function.

Statement 2.12 will be presented to participants in Survey 2.

RAMP CONSUMER DELPHI STUDY

Survey 2 Introduction

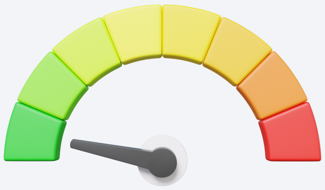

Statement 1.14: *I would be willing to participate in remote tests of my physical function (e.g., on a video call with a health professional, or by myself using written instructions and/or video demonstrations provided to me)*

Agreement: 79.5%

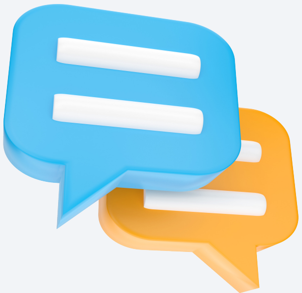

Moderate agreement for this statement was influenced primarily by concerns about access to and familiarity with technology, not believing it was necessary due to currently having good physical function, or preference for in-person assessments to ensure their safety and effectiveness.

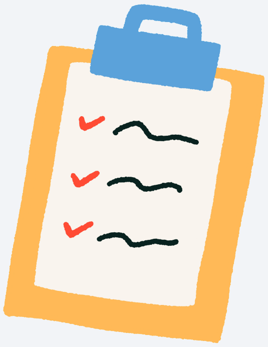

Statement 2.14: *If I felt I needed help to improve or maintain my physical function, I would be willing to participate in a remote test (e.g., supervised on a live video call with a health professional, unsupervised using printed instructions and/or video demonstrations provided to me etc)*

Statement 2.24*: I would be more likely to participate in a remote test of physical function if I was confident that the test was safe and accurate to perform by myself, and I had access to the necessary information and resources, including technology and equipment, to perform the test myself*

The revised statement 2.14 recognises that people are most likely to be open to remote tests if they have concerns about their physical function. The new statement 2.24 recognises that people require reassurance that remote tests are accessible, safe and accurate.

Statements 2.14 and 2.24 will be presented to participants in Survey 2.

RAMP CONSUMER DELPHI STUDY

Survey 2 Introduction

Statements 1.16, 1.17 and 1.18 are presented together on the following pages as they cover similar concepts

Statement 1.16: *I would be willing to participate in a remote exercise program to improve my physical function if it was ALWAYS supervised (e.g., exercising while on a live video call with a health professional for all exercise sessions)*

Agreement: 49.5%

Statement 1.17: *I would be willing to participate in a remote exercise program if it was SOMETIMES supervised (e.g., exercising on a live video call with a health professional for some exercise sessions, but exercising by myself unsupervised using instructions provided by the health professional for other sessions)*

Agreement: 63.4%

Statement 1.18: *I would be willing to participate in a remote exercise program if it was NOT supervised (e.g., exercising by myself unsupervised using instructions provided by a health professional)*

Agreement: 49.5%

Survey 2 Introduction

Low to moderate agreement for statements 1.16, 1.17 and 1.18 was influenced primarily by preferences for exercising under supervision or alone. Other reasons included not having current concerns about phyiscal function, and requiring access to technology and exercise equipment. Specific reasons for low agreement also included:

1.16 - not needing supervision; not wanting to be restricted to a set exercise schedule

1.17 and 1.18 - preference for constant supervision (or at least initial supervision) to ensure safety and motivation

Statement 2.16: *If I felt I needed help to improve or maintain my physical function, I would be willing to participate in a remote exercise program suited to my preferences at the time which may include exercise supervised by a health professional, and/or exercise led by myself*

Statement 2.25: *I would be more likely to participate in a remote exercise program if I was confident that I had access to the necessary information and resources, including technology and exercise equipment, to exercise safely and effectively*

The revised statement 2.16 captures concepts from 1.16, 1.17 and 1.18, and recognises that remote exercise programs should meet personal preferences regarding health professional support or independent management. The new statement 2.25 recognises that remote exercise programs require access to necessary resources.

Statements 2.16 and 2.25 will be presented to participants in Survey 2.

Survey 2 Introduction

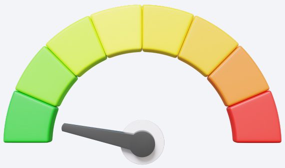

Statements 1.19 and 1.20 are presented together on the following pages as they cover similar concepts

Statement 1.19: *If I was to participate in a remote exercise program I would be happy to do so with a group (e.g., exercising by myself at home but while on a video call with other people like me who are also exercising at home, with or without the supervision of a health professional)*
Agreement: 45.3%

Statement 1.20: *If I was to participate in a remote exercise program to improve my physical function, I would be happy to do so alone without other people like me involved in the exercise sessions (e.g., exercising by myself at home with or without supervision by a health professional)*
Agreement: 77.3%

Survey 2 Introduction

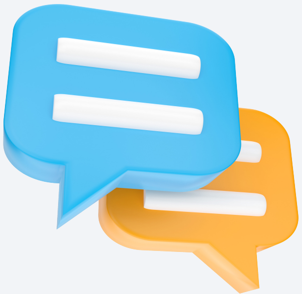

Low to moderate agreement for statements 1.19 and 1.20 was influenced primarily by differences in individual preferences for exercising alone or in group settings. Other reasons included preferences for in-person exercise sessions, requiring exercise supervision (at least initially), lack of access to resources, and safety concerns. Specific reasons for low agreement to each statement included:

1.19 - preference for solo exercise and not wanting other people to see me exercising; not wanting to be restricted to a set exercise schedule

1.20 - preference for a group setting to provide motivation and social connection; one-on-one exercise supervision more costly than group exercise.

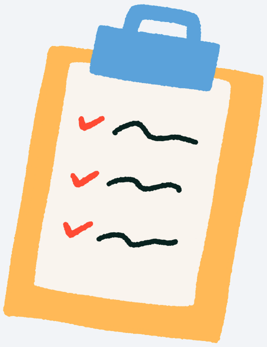

Statement 2.19: *If I felt I needed help to maintain or improve my physical function, I would be willing to participate in a remote exercise program suited to my preferences at the time which may include exercise performed by myself, and/or exercise performed with a group of people*

The revised statement 2.19 captures concepts from 1.19 and 1.20 and recognises that remote exercise programs should meet personal preferences regarding individual or group exercise. The new statement 2.25 (described earlier) also addresses concerns regarding access to resources that were raised in comments on 1.19 and 1.20.

Statement 2.19 will be presented to participants in Survey 2.

RAMP CONSUMER DELPHI STUDY

Survey 2 Summary

In additon to several other questions, you will be asked to rate your agreement with the following statements in Survey 2:

2.10: *I would like access to simple and reliable instructions on how to test my physical function myself so that I can monitor how it changes over time*

2.12: *If I felt I needed help to improve or maintain my physical function, having access to simple information about this (including advice on appropriate health professionals to discuss it with) would help me to have more informed conversations with health professionals about my physical function*

2.14: *If I felt I needed help to improve or maintain my physical function, I would be willing to participate in a remote test (e.g., supervised on a live video call with a health professional, unsupervised using printed instructions and/or video demonstrations provided to me etc)*

2.16: *If I felt I needed help to improve or maintain my physical function, I would be willing to participate in a remote exercise program suited to my preferences at the time which may include exercise supervised by a health professional, and/or exercise led by myself*

2.19: *If I felt I needed help to maintain or improve my physical function, I would be willing to participate in a remote exercise program suited to my preferences at the time which may include exercise performed by myself, and/or exercise performed with a group of people*

2.24: *I would be more likely to participate in a remote test of physical function if I was confident that the test was safe and accurate to perform by myself, and I had access to the necessary information and resources, including technology and equipment, to perform the test myself*

2.25: *I would be more likely to participate in a remote exercise program if I was confident that I had access to the necessary information and resources, including technology and exercise equipment, to exercise safely and effectively*
