# Supplementary material for: Developing Consumer Consensus on Remote Assessment and Management of Physical Function in Older Adults (RAMP): International Modified Delphi Process
Source: JMIR Aging. 2026 Feb 6;9:e75791. doi: 10.2196/75791 (PMC12924037; doi:10.2196/75791)
Supplement: Multimedia Appendix 4 [file aging_v9i1e75791_app4.pdf]

**Multimedia Appendix 4.** Summary of changes to Round 1 Delphi statements with moderate or low agreement.

| Round 1 Statement Number | Round 1 Statement | Reasons for moderate or low agreement | Round 2 Statement Number | Round 2 Statement |
|---|---|---|---|---|
| 1.10 | I would like access to information about how to test my physical function myself to determine if it is poor | • Some consumers wish to assess physical function even when it is not poor in order to monitor changes over time<br>• Some consumers require information that is reliable and easy to follow | 2.10 | I would like access to simple and reliable instructions on how to test my physical function myself so that I can monitor how it changes over time |
| 1.12 | Having better access to information on physical function would help me to have conversations about this with health professionals | • Some consumers feel that this is not required unless they are concerned about their physical function<br>• Some consumers feel their existing knowledge is adequate<br>• Some consumers feel that access to health professionals with relevant expertise is limited<br>• Some consumers feel that general practitioners do not have time or are not interested in discussing physical function<br>• Some consumers feel that they are already able to have conversations with health professionals | 2.12 | If I felt I needed help to improve or maintain my physical function, having access to simple information about this (including advice on appropriate health professionals to discuss it with) would help me to have more informed conversations with health professionals about my physical function |
| 1.14 | I would be willing to participate in remote tests of my physical function (e.g., on a video call with a health professional, or by myself using written instructions and/or video demonstrations provided to me) | • Some consumers reported a preference for in-person assessments in order to be confident in their safety and effectiveness *(this concept is addressed in new statement 2.24 below)*<br>• Some consumers feel that access to, and familiarity with, technology is a barrier | 2.14 | If I felt I needed help to improve or maintain my physical function, I would be willing to participate in a remote test (e.g., supervised on a live video call with a health professional, unsupervised using printed instructions and/or video demonstrations provided to me etc) |

| | | | | |
|---|---|---|---|---|
| | | to remote tests *(this concept is addressed in new statement 2.24 below)*<br>• Some consumers feel that remote tests are not necessary as they currently have acceptable physical function | | |
| 1.16 | I would be willing to participate in a remote exercise program to improve my physical function if it was ALWAYS supervised (e.g., exercising while on a live video call with a health professional for all exercise sessions) | • Across Statements 1.16, 1.17 and 1.18, consumers demonstrate differing preferences for participating in self-directed versus supervised exercise and acknowledge that these preferences can change over time *(this is addressed in statement 2.16 which incorporates the exercise approaches proposed in 1.16, 1.17 and 1.18)*<br>• Some consumers feel that remote exercise programs are not necessary as they currently have acceptable physical function<br>• Some consumers report that the requirement for access to technology and exercise equipment may be a barrier to remote exercise *(this concept is now addressed in new statement 2.25 below)* | 2.16 | If I felt I needed help to improve or maintain my physical function, I would be willing to participate in a remote exercise program suited to my preferences at the time which may include exercise supervised by a health professional, and/or exercise led by myself |
| 1.17 | I would be willing to participate in a remote exercise program if it was SOMETIMES supervised (e.g., exercising on a live video call with a health professional for some exercise sessions, but exercising by myself unsupervised using instructions provided by the health professional for other sessions) | | | |
| 1.18 | I would be willing to participate in a remote exercise program if it was NOT supervised (e.g., exercising by myself unsupervised using instructions provided by a health professional) | | | |
| 1.19 | If I was to participate in a remote exercise program I would be happy to do so with a group (e.g., exercising by myself at home but | • For Statements 1.19 and 1.20, consumers demonstrate differing preferences for participating in group | 2.19 | If I felt I needed help to maintain or improve my physical function, I would be willing to participate in a remote exercise program suited to |

| | | | | |
|---|---|---|---|---|
| | while on a video call with other people like me who are also exercising at home, with or without the supervision of a health professional) | versus individual exercise and acknowledge that these preferences can change over time *(this is addressed in statement 2.19 which incorporates the exercise approaches proposed in 1.19 and 1.20)*<br>• Some consumers report that the requirement for access to technology and exercise equipment may be a barrier to remote exercise *(this concept is now addressed in new statement 2.25 below)* | | my preferences at the time which may include exercise performed by myself, and/or exercise performed with a group of people |
| 1.20 | If I was to participate in a remote exercise program to improve my physical function, I would be happy to do so alone without other people like me involved in the exercise sessions (e.g., exercising by myself at home with or without supervision by a health professional) | | | |
| - | - | • A common theme across statements in Round 1 is that some consumers would value reassurance that remote tests are accessible, safe and accurate | 2.24 | I would be more likely to participate in a remote test of physical function if I was confident that the test was safe and accurate to perform by myself, and I had access to the necessary information and resources, including technology and equipment, to perform the test myself |
| - | - | • A common theme across statements in Round 1 is that some consumers acknowledge that remote exercise programs require access to appropriate information and resources, including technology and exercise equipment | 2.25 | I would be more likely to participate in a remote exercise program if I was confident that I had access to the necessary information and resources, including technology and exercise equipment, to exercise safely and effectively |
