# Supplementary material for: Developing Consumer Consensus on Remote Assessment and Management of Physical Function in Older Adults (RAMP): International Modified Delphi Process
Source: JMIR Aging. 2026 Feb 6;9:e75791. doi: 10.2196/75791 (PMC12924037; doi:10.2196/75791)
Supplement: Multimedia Appendix 5 [file aging_v9i1e75791_app5.pdf]

RAMP Consumer Delphi Survey 2

Start of Block: Plain Language Summary

Q1.1

Thank you for contributing to the second survey in this important study on physical function in older adults. As a reminder, a summary of the study's purpose is provided below:

What is physical function?
'Physical function' is the ability of a person to perform everyday activities. Such activities may include walking, climbing stairs, carrying shopping bags, preparing meals, and cleaning your house. Having good physical function allows people to perform everyday activities so that they can stay independent and enjoy a good quality of life.

Having **poor physical function** can limit a person's ability to move about their home and community, participate in work, hobbies, and spend time with family and friends. Poor physical function can be caused by factors including low physical activity (e.g. bed rest), chronic diseases (e.g. heart disease), illnesses (e.g. flu), or injuries (e.g. a breaking a bone).

However, the group most commonly affected by poor physical function is adults aged 60 years or older. This is because as we get older, our muscles get weaker, making it more difficult to move around and do tasks that require muscle strength.

Unfortunately, poor physical function is not regularly diagnosed or treated by health professionals (e.g. doctors, nurses, physiotherapists, exercise physiologists etc). Many people are also unaware that there are things they can do themselves to improve their own physical function.

What is this study about?
The aim of this study is to understand how important physical function is to people aged 60 years and older. We also hope to understand how people would like to receive support from health professionals to address concerns about their physical function. Finally, we would like to understand what aspects of physical function older adults might be able to address themselves.

Please click the right arrow below to continue the survey. You can also use the left arrow at any time to go back to a previous page of the survey.

Page Break

Q50

This study uses a method known as a "Delphi process". This involves two separate surveys aimed at achieving agreement among participants on important issues related to physical function.

You have been invited to complete this current survey, which is the second and final survey in our Delphi process, as you have already completed the first survey (between August and November 2023).

The findings from the first survey have been analysed and a summary of the results can be viewed by clicking here: Survey 1 Results Summary

We strongly encourage you to read this document as it will help you to understand the views of your fellow study participants in Survey 1 and also what is included in Survey 2. This second survey generally aims to resolve areas of disageement that were identified in Survey 1.

It is important that you complete Survey 2 because understanding the wide range of opinions allows us to develop recommendations for supporting older adults to maintain and improve their physical function.

Completing the survey

Your participation is voluntary and anonymous, and if you have any questions or concerns, please email the principal researcher Associate Professor David Scott (d.scott@deakin.edu.au).

In this online survey, you will be asked to respond to a range of questions and statements. The survey should take approximately 10 to 15 minutes to complete.

You will be asked questions about yourself and your opinions about physical function. Some of these questions are identical or similar to questions you responded to in Survey 1. For some questions, you will be asked to choose from a range of responses. For others, you will be asked to rate your level of agreement with a given statement. You can add comments to your responses to provide us with more information if you wish to do so.

End of Block: Plain Language Summary

Start of Block: Eligibility

Q140 **Please enter your email address below.**

____

Page Break

Q57 Please select your **current age (years)** from the drop-down list below (note only people aged 60 years or older can participate in this study):

▼ 60 (1) ... 120 (61)

Q56 Please click the "I'm not a robot" box below, followed by the right arrow.

End of Block: Eligibility

Start of Block: Demographics Questions

Q3.3 **What is your gender?**

- ◯ Male (1)
- ◯ Female (2)
- ◯ Non-binary / third gender (3)
- ◯ Prefer not to say (4)

X→

Q4.1 **Please select the *country you currently live in* from the drop-down list below:**

▼ Afghanistan (1) ... Zimbabwe (1357)

Q3.8 **Would you say that in general your health is (please tick):**

- ○ Excellent (1)
- ○ Very Good (2)
- ○ Good (3)
- ○ Fair (4)
- ○ Poor (5)

End of Block: Demographics Questions

Start of Block: Current Physical Function and Experiences Questions

Q8.1 **Compared with when you were 40 years old, would you say your physical function is *currently*:**

- ○ Much worse (1)
- ○ Somewhat worse (2)
- ○ Neither better nor worse (3)
- ○ Somewhat better (4)
- ○ Much better (5)

Q141 **Compared with when you completed the first survey in this study (between August and November 2023), would you say your physical function is *currently*:**

- ○ Much worse (1)
- ○ Somewhat worse (2)
- ○ Neither better nor worse (3)
- ○ Somewhat better (4)
- ○ Much better (5)

Q5.1 **How much difficulty do you have in lifting and carrying 10 pounds / 4.5 kilograms (e.g. about the weight of an ironing board or two normal-sized house bricks)?**

- ○ None at all (1)
- ○ Some (2)
- ○ A lot or unable (3)

Q5.2 **How much difficulty do you have walking across a room?**

- ○ None at all (1)
- ○ Some (2)
- ○ A lot, use walking aids (e.g. walking frame or walking stick), or unable (3)

Q5.3 **How much difficulty do you have transferring (i.e., getting up) from a chair or bed?**

- ○ None at all (1)
- ○ Some (2)
- ○ A lot or unable (3)

Q5.4 **How much difficulty do you have climbing a flight of 10 stairs?**

- ○ None at all (1)
- ○ Some (2)
- ○ A lot or unable (3)

Q5.5 **How many times have you fallen in the past year?**

- ○ None at all (1)
- ○ 1 - 3 falls (2)
- ○ 4 or more falls (3)

Page Break

End of Block: Current Physical Function and Experiences Questions

Start of Block: Physical Function Statements

Q8.2 Please rate your level of agreement with the following statements on physical function, where: *0 = strongly disagree,* *5 = neither agree nor disagree,* *10 = strongly agree.* To respond to each statement, *drag the slider* to the number that best corresponds with your level of agreement/disagreement.

I would like access to simple and reliable instructions on how to test my physical function myself so that I can monitor how it changes over time:

	0	1	2	3	4	5	6	7	8	9	10
0 = Strongly disagree; 5 = neither agree nor disagree; 10 = Strongly agree ()											

Q80 You may provide further details if you wish:

____

Q81 **If I felt I needed help to improve or maintain my physical function, having access to simple information about this (including advice on appropriate health professionals to discuss it with) would help me to have more informed conversations with health professionals about my physical function:**

	0	1	2	3	4	5	6	7	8	9	10
0 = Strongly disagree; 5 = neither agree nor disagree; 10 = Strongly agree ()											

Q82 You may provide further details if you wish:

____

Q83 **If I felt I needed help to improve or maintain my physical function, I would be willing to participate in a remote test (e.g., supervised on a live video call with a health professional, unsupervised using printed instructions and/or video demonstrations provided to me etc):**

	0	1	2	3	4	5	6	7	8	9	10
0 = Strongly disagree; 5 = neither agree nor disagree; 10 = Strongly agree ()											

Q84 You may provide further details if you wish:

____

Q85 **If I felt I needed help to improve or maintain my physical function, I would be willing to participate in a remote exercise program suited to my preferences at the time which may include exercise supervised by a health professional, and/or exercise led by myself:**

	0	1	2	3	4	5	6	7	8	9	10
0 = Strongly disagree; 5 = neither agree nor disagree; 10 = Strongly agree ()											

Q144 You may provide further details if you wish:

____

Q85b **If I felt I needed help to maintain or improve my physical function, I would be willing to participate in a remote exercise program suited to my preferences at the time which may include exercise performed by myself, and/or exercise performed with a group of people:**

	0	1	2	3	4	5	6	7	8	9	10
0 = Strongly disagree; 5 = neither agree nor disagree; 10 = Strongly agree ()											

Q86 You may provide further details if you wish:

____

Q142 **I would be more likely to participate in a remote test of physical function if I was confident that the test was safe and accurate to perform by myself, and I had access to the necessary information and resources, including technology and equipment, to perform the test myself:**

	0	1	2	3	4	5	6	7	8	9	10
0 = Strongly disagree; 5 = neither agree nor disagree; 10 = Strongly agree ()											

Q145 You may provide further details if you wish:

____

Q143 **I would be more likely to participate in a remote exercise program if I was confident that I had access to the necessary information and resources, including technology and exercise equipment, to exercise safely and effectively:**

	0	1	2	3	4	5	6	7	8	9	10
0 = Strongly disagree; 5 = neither agree nor disagree; 10 = Strongly agree ()											

Q85b.2 You may provide further details if you wish:

____

End of Block: Physical Function Statements

Start of Block: Close Survey

Q7.7 **Do you have any other thoughts or concerns about remote assessment and management of your physical function that you would like to share?**

____

Q122

You have reached the end of this survey. Thank you for your contribution to the RAMP Delphi process!

What happens next?

Once this survey closes, the research team will begin conducting an analysis of participants' responses and then prepare a summary of the final results of this Delphi process. These will be shared with you via your email address in the coming months.

If you have any questions or comments in the meantime, please contact Associate Professor David Scott (d.scott@deakin.edu.au).

Please click the right arrow below to submit your responses.

End of Block: Close Survey
