# Supplementary material for: Developing Consumer Consensus on Remote Assessment and Management of Physical Function in Older Adults (RAMP): International Modified Delphi Process
Source: JMIR Aging. 2026 Feb 6;9:e75791. doi: 10.2196/75791 (PMC12924037; doi:10.2196/75791)
Supplement: Multimedia Appendix 7 [file aging_v9i1e75791_app7.pdf]

Multimedia Appendix 3. Delivery preferences and perceived positives and negatives of remote tests and treatments for physical function among RAMP participants in Round 1.

	**N (%)**
**Which of the below methods would you be willing to use in order to participate in remote tests and treatments for physical function (Select all that apply)?**	
Written documents (e.g. flyers, brochures, magazines, books etc)	444 (68.9)
Telephone calls	296 (46.0)
Video calls (e.g. FaceTime, Zoom etc)	430 (66.8)
Emails or text messages	413 (64.1)
Videos (e.g. on a website or DVD)	500 (77.6)
Websites	445 (69.1)
Smartphone or tablet applications	424 (65.8)
Other	24 (3.7)
None of the above (I would not be willing to participate in remote tests and treatments for physical function)	32 (5.0)
**Which of the below factors do you perceive as potential positives of remote tests and treatments for physical function? (Select all that apply)**	
Convenience of not needing to travel to appointments	566 (87.9)
Flexibility to perform assessments and exercises when convenient	518 (80.4)
Ability to complete tasks at my own pace	481 (74.7)
Lower waiting times to see a health professional	438 (68.0)
Lower cost compared to attending in-person sessions	414 (64.3)
Ability to access health professionals who would otherwise be unavailable	365 (56.7)
Other	24 (3.7)
**Which of the below factors do you perceive as potential negatives of remote tests and treatments for physical function? (Select all that apply)**	
Lack of personalised guidance during exercises	328 (50.9)
Performing tests and exercises without supervision in my home may not be effective	273 (42.4)
Performing tests and exercises without supervision in my home may be unsafe	128 (19.9)
Lack of social interaction and motivation from others	264 (41.0)
Difficulty using technology	114 (17.7)
Concerns about privacy and security when using technology	99 (15.4)
Other	93 (14.4)
